# Higher COVID-19 pneumonia risk associated with anti-IFN-α than with anti-IFN-ω auto-Abs in children

**DOI:** 10.1084/jem.20231353

**Published:** 2024-01-04

**Authors:** Paul Bastard, Adrian Gervais, Maki Taniguchi, Liisa Saare, Karita Särekannu, Tom Le Voyer, Quentin Philippot, Jérémie Rosain, Lucy Bizien, Takaki Asano, Marina Garcia-Prat, Alba Parra-Martínez, Mélanie Migaud, Miyuki Tsumura, Francesca Conti, Alexandre Belot, Jacques G. Rivière, Tomohiro Morio, Junko Tanaka, Etienne Javouhey, Filomeen Haerynck, Sotirija Duvlis, Tayfun Ozcelik, Sevgi Keles, Yacine Tandjaoui-Lambiotte, Simon Escoda, Maya Husain, Qiang Pan-Hammarström, Lennart Hammarström, Gloria Ahlijah, Anthony Abi Haidar, Camille Soudee, Vincent Arseguel, Hassan Abolhassani, Sabina Sahanic, Ivan Tancevski, Yoko Nukui, Seiichi Hayakawa, George P. Chrousos, Athanasios Michos, Elizabeth-Barbara Tatsi, Filippos Filippatos, Agusti Rodriguez-Palmero, Jesus Troya, Imran Tipu, Isabelle Meyts, Lucie Roussel, Sisse Rye Ostrowski, Laire Schidlowski, Carolina Prando, Antonio Condino-Neto, Nathalie Cheikh, Ahmed A. Bousfiha, Jalila El Bakkouri, COVID Clinicians, Sergio Aguilera-Albesa, Suzan A. AlKhater, Gulsum Alkan, Riccardo Castagnoli, Cyril Cyrus, Sefika Elmas Bozdemir, Melike Emiroglu, Belgin Gulhan, Emine Hafize Erdeniz, Nevin Hatipoglu, Gülsün Iclal Bayhan, Petr Jabandziev, Saliha Kanik Yuksek, Adem Karbuz, Şadiye Kübra Tüter Öz, Gian Luigi Marseglia, Ozge Metin Akcan, Ahmet Osman Kılıç, Aslinur Ozkaya Parlakay, Maria Papadaki, Katerina Slaba, Esra Sevketoglu, Juan Valencia-Ramos, Aysun Yahşi, GEN-COVID Study Group, Antonio Aguilera Guirao, Julián Álvarez Escudero, Antonio Antela López, Gema Barbeito Castiñeiras, Xabier Bello Paderne, Miriam Ben García, María Victoria Carral García, Miriam Cebey López, Amparo Coira Nieto, Mónica Conde Pájaro, José Javier Costa Alcalde, María José Currás Tuala, Ana Isabel Dacosta Urbieta, Blanca Díaz Esteban, María Jesús Domínguez Santalla, Cristina Fernández Pérez, Juan Fernández Villaverde, Cristóbal Galbán Rodríguez, José Luis García Allut, Luisa García Vicente, Elena Giráldez Vázquez, Alberto Gómez Carballa, José Gómez Rial, Francisco Javier González Barcala, Beatriz Guerra Liñares, Pilar Leboráns Iglesias, Beatriz Lence Massa, Marta Lendoiro Fuentes, Montserrat López Franco, Ana López Lago, Federico Martinón-Torres, Antonio Salas, Daniel Navarro De la Cruz, Eloína Núñez Masid, Juan Bautista Ortolá Devesa, Jacobo Pardo Seco, María Pazo Núñez, Marisa Pérez del Molino Bernal, Hugo Pérez Freixo, Lidia Piñeiro Rodríguez, Sara Pischedda, Manuel Portela Romero, Antonio Pose Reino, Gloria María Prada Hervella, Teresa Queiro Verdes, Lorenzo Redondo Collazo, Patricia Regueiro Casuso, Susana Rey García, Sara Rey Vázquez, Vanessa Riveiro Blanco, Irene Rivero Calle, Carmen Rivero Velasco, Nuria Rodríguez Núñez, Carmen Rodríguez-Tenreiro Sánchez, Eva Saborido Paz, José Miguel Sadiki Orayyou, Carla Saito Villanueva, Sonia Serén Fernández, Pablo Souto Sanmartín, Manuel Taboada Muñiz, Rocío Trastoy Pena, Mercedes Treviño Castellano, Luis Valdés Cuadrado, Pablo Varela García, María Soledad Vilas Iglesias, Sandra Viz Lasheras, Rocio Ferreiro-Iglesias, Iria Bastón-Rey, Cristina Calviño-Suárez, COVID Human Genetic Effort, Laurent Abel, Alessandro Aiuti, Saleh Al-Muhsen, Fahd Al-Mulla, Ali Amara, Mark S. Anderson, Evangelos Andreakos, Andrés A. Arias, Lisa M. Arkin, Hagit Baris Feldman, Paul Bastard, Alexandre Belot, Catherine M. Biggs, Dusan Bogunovic, Alexandre Bolze, Anastasiia Bondarenko, Ahmed A. Bousfiha, Petter Brodin, Yenan Bryceson, Manish J. Butte, Jean-Laurent Casanova, Giorgio Casari, John Christodoulou, Aurélie Cobat, Roger Colobran, Antonio Condino-Neto, Stefan N. Constantinescu, Megan A. Cooper, Clifton L. Dalgard, Murkesh Desai, Beth A. Drolet, Xavier Duval, Jamila El Baghdadi, Philippine Eloy, Sara Espinosa-Padilla, Jacques Fellay, Carlos Flores, José Luis Franco, Antoine Froidure, Guy Gorochov, Peter K. Gregersen, Bodo Grimbacher, Filomeen Haerynck, David Hagin, Rabih Halwani, Lennart Hammarström, James R. Heath, Elena W.Y. Hsieh, Eystein Husebye, Kohsuke Imai, Yuval Itan, Erich D. Jarvis, Emmanuelle Jouanguy, Elżbieta Kaja, Timokratis Karamitros, Kai Kisand, Cheng-Lung Ku, Yu-Lung Lau, Yun Ling, Carrie L. Lucas, Davood Mansouri, László Maródi, France Mentré, Isabelle Meyts, Joshua D. Milner, Kristina Mironska, Trine H. Mogensen, Tomohiro Morio, Lisa F.P. Ng, Luigi D. Notarangelo, Antonio Novelli, Giuseppe Novelli, Cliona O’Farrelly, Satoshi Okada, Keisuke Okamoto, Tayfun Ozcelik, Qiang Pan-Hammarström, Jean W. Pape, Rebeca Perez de Diego, Jordi Perez-Tur, David S. Perlin, Graziano Pesole, Anna M. Planas, Carolina Prando, Aurora Pujol, Anne Puel, Lluis Quintana-Murci, Sathishkumar Ramaswamy, Laurent Renia, Igor Resnick, Carlos Rodríguez-Gallego, Vanessa Sancho-Shimizu, Anna Sediva, Mikko R.J. Seppänen, Mohammed Shahrooei, Anna Shcherbina, Ondrej Slaby, Andrew L. Snow, Pere Soler-Palacín, Vassili Soumelis, András N. Spaan, Helen C. Su, Ivan Tancevski, Stuart G. Tangye, Ahmad Abou Tayoun, Şehime Gülsün Temel, Christian Thorball, Pierre Tiberghien, Sophie Trouillet-Assant, Stuart E. Turvey, K.M. Furkan Uddin, Mohammed J. Uddin, Diederik van de Beek, Donald C. Vinh, Horst von Bernuth, Joost Wauters, Mayana Zatz, Pawel Zawadzki, Qian Zhang, Shen-Ying Zhang, Pärt Peterson, Aurora Pujol, Romain Lévy, Pierre Quartier, Donald C. Vinh, Bertrand Boisson, Vivien Béziat, Shen-Ying Zhang, Alessandro Borghesi, Andrea Pession, Evangelos Andreakos, Nico Marr, Alexios-Fotios A. Mentis, Trine H. Mogensen, Carlos Rodríguez-Gallego, Pere Soler-Palacin, Roger Colobran, Vallo Tillmann, Bénédicte Neven, Sophie Trouillet-Assant, Petter Brodin, Laurent Abel, Emmanuelle Jouanguy, Qian Zhang, Federico Martinón-Torres, Antonio Salas, Alberto Gómez-Carballa, Luis I. Gonzalez-Granado, Kai Kisand, Satoshi Okada, Anne Puel, Aurélie Cobat, Jean-Laurent Casanova

**Affiliations:** 1 Laboratory of Human Genetics of Infectious Diseases, Necker Branch, INSERM U1163, Necker Hospital for Sick Children, Paris, France; 2 University Paris Cité, Imagine Institute, Paris, France; 3 St. Giles Laboratory of Human Genetics of Infectious Diseases, Rockefeller Branch, The Rockefeller University, New York, NY, USA; 4 Pediatric Hematology-Immunology and Rheumatology Unit, Necker Hospital for Sick Children, Assistance Publique-Hôpitaux de Paris (AP-HP), Paris, France; 5 Dept. of Pediatrics, Graduate School of Biomedical and Health Sciences, Hiroshima University, Hiroshima, Japan; 6 Dept. of Pediatrics, Institute of Clinical Medicine, University of Tartu, Tartu, Estonia; 7 Molecular Pathology, Institute of Biomedicine and Translational Medicine, University of Tartu, Tartu, Estonia; 8 Pediatric Infectious Diseases and Immunodeficiencies Unit, Hospital Universitari Vall d’Hebron, Vall d’Hebron Research Institute, Vall d’Hebron Barcelona Hospital Campus, Universitat Autònoma de Barcelona (UAB), Barcelona, Spain; 9 Pediatric Unit, IRCCS Azienda Ospedaliero-Universitaria di Bologna, Bologna, Italy; 10 Dept. of Medical and Surgical Sciences, Alma Mater Studiorum, University of Bologna, Bologna, Italy; 11 National Reference Center for Rheumatic, and Autoimmune and Systemic Diseases in Children, Lyon, France; 12 Immunopathology Federation LIFE, Hospices Civils de Lyon, Lyon, France; 13 Hospices Civils de Lyon, Lyon, France; 14 International Center of Research in Infectiology, Lyon University, International Center of Research in Infectiology, Lyon University, INSERM U1111, CNRS UMR 5308, ENS, UCBL, Lyon, France; 15 Dept. of Pediatrics and Developmental Biology, Graduate School of Medical and Dental Sciences, Tokyo Medical and Dental University (TMDU), Tokyo, Japan; 16 Dept. of Epidemiology, Infectious Disease Control and Prevention, Graduate School of Biomedical and Health Sciences, Hiroshima University, Hiroshima, Japan; 17 Pediatric Intensive Care Unit, Hospices Civils de Lyon, Hopital Femme Mère Enfant, Lyon, France; 18 Dept. of Paediatric Immunology and Pulmonology, Center for Primary Immunodeficiency Ghent, Jeffrey Modell Diagnosis and Research Center, Ghent University Hospital, Ghent, Belgium; 19 Faculty of Medical Sciences, University “Goce Delchev”, Stip, Republic of Northern Macedonia; 20 Institute of Public Health of the Republic of North Macedonia, Skopje, North Macedonia; 21 Dept. of Molecular Biology and Genetics, Bilkent University, Ankara, Turkey; 22 Meram Medical Faculty, Necmettin Erbakan University, Konya, Turkey; 23 Pulmonology and Infectious Disease Department, Saint Denis Hospital, Saint Denis, France; 24 INSERM UMR 1137 IAME, Paris, France; 25 INSERM UMR 1272 Hypoxia and Lung, Bobigny, France; 26 Pediatric Dept., Saint-Denis Hospital, Saint-Denis, France; 27 Division of Immunology, Dept. of Medical Biochemistry and Biophysics, Karolinska Institutet, Stockholm, Sweden; 28 Research Center for Immunodeficiencies, Pediatrics Center of Excellence, Children’s Medical Center, Pediatrics Center of Excellence, Tehran University of Medical Sciences, Tehran, Iran; 29 Dept. of Internal Medicine II, Medical University of Innsbruck, Innsbruck, Austria; 30 Dept. of Infection Control and Prevention, Medical Hospital, TMDU, Tokyo, Japan; 31 University Research Institute of Maternal and Child Health and Precision Medicine, National and Kapodistrian University of Athens, Athens, Greece; 32 First Dept. of Pediatics, National and Kapodistrian University of Athens, Athens, Greece; 33 Neurometabolic Diseases Laboratory, Bellvitge Biomedical Research Institute (IDIBELL), L’Hospitalet de Llobregat, Barcelona, Spain; 34 Dept. of Pediatrics, Germans Trias i Pujol University Hospital, UAB, Barcelona, Spain; 35 Centro de Investigación Biomédica en Red de Enfermedades Raras (CIBERER), Madrid, Spain; 36 Dept. of Internal Medicine, Infanta Leonor University Hospital, Madrid, Spain; 37 University of Management and Technology, Lahore, Pakistan; 38 Dept. of Immunology, Laboratory of Inborn Errors of Immunity, Microbiology and Transplantation, KU Leuven, Leuven, Belgium; 39 Dept. of Pediatrics, Jeffrey Modell Diagnostic and Research Network Center, University Hospitals Leuven, Leuven, Belgium; 40 Dept. of Medicine, Division of Infectious Diseases, McGill University Health Centre, Montréal, Canada; 41 Infectious Disease Susceptibility Program, Research Institute–McGill University Health Centre, Montréal, Canada; 42 Dept. of Clinical Immunology, Rigshospitalet, Copenhagen University Hospital, Copenhagen, Denmark; 43 Faculdades Pequeno Príncipe, Instituto de Pesquisa Pelé Pequeno Príncipe, Curitiba, Brazil; 44 Dept. of Immunology, Institute of Biomedical Sciences, University of São Paulo, São Paulo, Brazil; 45 Pediatric Hematology Unit, University Hospital of Besançon, Besançon, France; 46 Dept. of Pediatric Infectious Disease and Clinical Immunology, CHU Ibn Rushd and LICIA, Laboratoire d’Immunologie Clinique, Inflammation et Allergie, Faculty of Medicine and Pharmacy, Hassan II University, Casablanca, Morocco; 47 Laboratory of Immunology, CHU Ibn Rushd and LICIA, Laboratoire d’Immunologie Clinique, Inflammation et Allergie, Faculty of Medicine and Pharmacy, Hassan II University, Casablanca, Morocco; 48 Neurometabolic Diseases Laboratory, IDIBELL-Hospital Duran i Reynals, CIBERER U759, and Catalan Institution of Research and Advanced Studies, Barcelona, Spain; 49 Neonatal Intensive Care Unit, Fondazione IRCCS Policlinico San Matteo, Pavia, Italy; 50 Center for Clinical, Experimental Surgery and Translational Research, Biomedical Research Foundation of the Academy of Athens, Athens, Greece; 51 Research Branch, Sidra Medicine, Doha, Qatar; 52 Dept. of Infectious Diseases, Aarhus University Hospital, Skejby, Denmark; 53 Dept. of Biomedicine, Aarhus University, Aarhus, Denmark; 54 Hospital Universitario de Gran Canaria Dr Negrín, Canarian Health System, Las Palmas, Spain; 55 Dept. of Clinical Sciences, University Fernando Pessoa Canarias, Las Palmas de Gran Canaria, Spain; 56 Dept. of Medical and Surgical Sciences, School of Medicine, University of Las Palmas de Gran Canaria, Las Palmas de Gran Canaria, Spain; 57 Immunology Division, Genetics Dept., Hospital Universitari Vall d’Hebron, Vall d’Hebron Research Institute, Vall d’Hebron Barcelona Hospital Campus, UAB, Barcelona, Spain; 58 Joint Research Unit, Hospices Civils de Lyon-bio Mérieux, Hospices Civils de Lyon, Lyon Sud Hospital, Pierre-Bénite, France; 59 International Center of Research in Infectiology, Lyon University, INSERM U1111, CNRS UMR 5308, ENS, UCBL, Lyon, France; 60 Unit for Clinical Pediatrics, Dept. of Women’s and Children’s Health, Karolinska Institutet, Solna, Sweden; 61 Department of Immunology and Inflammation, Imperial College London, London, UK; 62 Translational Pediatrics and Infectious Diseases, Pediatrics Dept., Hospital Clínico Universitario de Santiago, Servizo Galego de Saude (SERGAS), Santiago de Compostela, Spain; 63 GENVIP Research Group, Instituto de Investigación Sanitaria de Santiago (IDIS), Universidad de Santiago de Compostela, Galicia, Spain; 64 Centro de Investigación Biomédica en Red de Enfermedades Respiratorias, Instituto de Salud Carlos III, Madrid, Spain; 65 Facultade de Medicina, Unidade de Xenética, Instituto de Ciencias Forenses, Universidade de Santiago de Compostela, and GenPoB Research Group, IDIS, SERGAS, Galicia, Spain; 66 Immunodeficiencies Unit, Hospital 12 de octubre, Research Institute Hospital 12 octubre, Madrid, Spain; 67 Howard Hughes Medical Institute, New York, NY, USA; 68 Dept. of Pediatrics, Necker Hospital for Sick Children, AP-HP, Paris, France

## Abstract

We found that 19 (10.4%) of 183 unvaccinated children hospitalized for COVID-19 pneumonia had autoantibodies (auto-Abs) neutralizing type I IFNs (IFN-α2 in 10 patients: IFN-α2 only in three, IFN-α2 plus IFN-ω in five, and IFN-α2, IFN-ω plus IFN-β in two; IFN-ω only in nine patients). Seven children (3.8%) had Abs neutralizing at least 10 ng/ml of one IFN, whereas the other 12 (6.6%) had Abs neutralizing only 100 pg/ml. The auto-Abs neutralized both unglycosylated and glycosylated IFNs. We also detected auto-Abs neutralizing 100 pg/ml IFN-α2 in 4 of 2,267 uninfected children (0.2%) and auto-Abs neutralizing IFN-ω in 45 children (2%). The odds ratios (ORs) for life-threatening COVID-19 pneumonia were, therefore, higher for auto-Abs neutralizing IFN-α2 only (OR [95% CI] = 67.6 [5.7–9,196.6]) than for auto-Abs neutralizing IFN-ω only (OR [95% CI] = 2.6 [1.2–5.3]). ORs were also higher for auto-Abs neutralizing high concentrations (OR [95% CI] = 12.9 [4.6–35.9]) than for those neutralizing low concentrations (OR [95% CI] = 5.5 [3.1–9.6]) of IFN-ω and/or IFN-α2.

## Teaser

Auto-Abs neutralizing IFN-α and/or IFN-ω are found in about 10% of children with COVID-19 pneumonia. In uninfected children, auto-Abs neutralizing IFN-α2 are rarer (0.2%) than those neutralizing IFN-ω (2%). Auto-Abs against IFN-α thus confer a greater risk of COVID-19 pneumonia.

## Introduction

Since the start of the pandemic of coronavirus disease 19 (COVID-19) (Zhou et al., 2020), caused by severe respiratory syndrome coronavirus 2 (SARS-CoV-2), close to 7 million people have died from COVID-19 pneumonia (Worldometers, 2023). Age is the major epidemiological risk factor for death from pneumonia in unvaccinated individuals, with the risk doubling every 5 years of age from childhood onward (Bogunovic and Merad, 2021; Levin et al., 2020; O’Driscoll et al., 2021). Unvaccinated adults with inborn errors of immunity (IEI) affecting the production of, or response to, type I IFNs, or both, are prone to critical COVID-19 pneumonia (Asano et al., 2021; Khanmohammadi et al., 2021; Zhang et al., 2020b). These findings established the crucial role of type I IFNs in fending off SARS-CoV-2 and explained about 1–5% of cases (Zhang et al., 2022a). Autoantibodies (auto-Abs) neutralizing high concentrations (10 ng/ml in plasma diluted 1/10) of IFN-α2 and/or IFN-ω were found in at least another 10% of unvaccinated adults with critical COVID-19 pneumonia (Bastard et al., 2020). This observation was later replicated in various regions of the world (Abers et al., 2021; Acosta-Ampudia et al., 2021; Akbil et al., 2022; Arrestier et al., 2022; Bastard et al., 2021c; Busnadiego et al., 2022; Carapito et al., 2022; Chang et al., 2021; Chauvineau-Grenier et al., 2022a; Chauvineau-Grenier et al., 2022b; Credle et al., 2022; Eto et al., 2022; Frasca et al., 2022; Goncalves et al., 2021; Grimm et al., 2023; Hansen et al., 2023; Koning et al., 2021; Lamacchia et al., 2022; Lemarquis et al., 2021; Mathian et al., 2022; Meisel et al., 2021; Petrikov et al., 2022; Philippot et al., 2023; Pons et al., 2023; Raadsen et al., 2022; Savvateeva et al., 2021; Schidlowski et al., 2022; Simula et al., 2022; Solanich et al., 2021; Soltani-Zangbar et al., 2022; Su et al., 2022; Troya et al., 2021; van der Wijst et al., 2021; Vanker et al., 2023; Vazquez et al., 2021; Wang et al., 2021; Ziegler et al., 2021). Moreover, at least 13% of unvaccinated adults with critical COVID-19 pneumonia were found to have auto-Abs neutralizing lower, more physiological concentrations (100 pg/ml in plasma diluted 1/10) of IFN-α2 and/or IFN-ω, whereas auto-Abs neutralizing IFN-β (10 ng/ml in plasma diluted 1/10) were found in another 1% of patients (Bastard et al., 2021a).

These auto-Abs collectively account for about 20% of COVID-19 deaths across age groups in adults (Bastard et al., 2021a; Manry et al., 2022). They are present before infection and are causal for critical disease, being second only to age in importance as a risk factor (Manry et al., 2022). Remarkably, the prevalence of these auto-Abs in the adult general population remains stable until the age of 70 years (about 0.3% for auto-Abs neutralizing high concentrations of IFN and 1% for auto-Abs neutralizing low concentrations of IFNs), after which it increases sharply (reaching up to 4% and 7%, respectively, in individuals aged 80–85 years), consistent with the higher risk of life-threatening COVID-19 in the elderly population (Bastard et al., 2021a; Manry et al., 2022). Finally, the presence of these auto-Abs has been reported in about 20% of adults suffering from “breakthrough” hypoxemic COVID-19 pneumonia despite an appropriate Ab response to two injections of RNA vaccine (Bastard et al., 2022; Sokal et al., 2023). They also underlie 5% and 20% of cases of critical influenza and Middle East respiratory syndrome (MERS) pneumonia (Alotaibi et al., 2023; Zhang et al., 2022c), respectively, a third of the rare life-threatening adverse reactions to yellow fever vaccination (Bastard et al., 2021b), and about 40% of cases of West Nile virus encephalitis (Gervais et al., 2023), while contributing to herpetic viral infections in various contexts (Pozzetto et al., 1984; Nagafuchi et al., 2007; Hetemäki et al., 2021b; Mathian et al., 2022). Overall, auto-Abs against type I IFNs can underlie a significant number of cases of severe viral diseases in adults (Bucciol et al., 2023; Casanova and Anderson, 2023; Cobat et al., 2023; Hale, 2023; Quiros-Roldan et al., 2023; Samuel, 2023; Su et al., 2023; Tangye et al., 2023).

These genetic and immunological deficits account for about 20% of cases of critical COVID-19 pneumonia in adults. They provide a general mechanism for pathogenesis of the disease in adults, with insufficient type I IFN immunity during the first days of infection being the key driver of the disease (Campbell et al., 2022; Casanova and Abel, 2021, 2022; Casanova and Anderson, 2023; Cobat et al., 2023; Garcia-Garcia et al., 2023; Samuel, 2023; Su et al., 2023; Zhang et al., 2020a; Zhang et al., 2022a). However, much less is known about life-threatening COVID-19 pneumonia in children. Children are very rarely hospitalized for COVID-19 pneumonia, with the risk of hospitalization being only about 0.1% (O’Driscoll et al., 2021). Recessive inborn errors underlying complete deficiencies of a small set of genes governing type I IFN immunity have been found in ∼10% of an international cohort of children hospitalized for COVID-19 pneumonia (COVID Human Genetic Effort [CHGE]; https://www.covidhge.com), suggesting that the same mechanisms of disease are at work in adults and children (Zhang et al., 2022b). Most children with X-linked recessive TLR7 deficiency, or autosomal recessive IFNAR1, TBK1, STAT2, or TYK2 deficiency infected with SARS-CoV-2 suffered life-threatening COVID-19 pneumonia (Asano et al., 2021; Schmidt et al., 2021; Zhang et al., 2022b). However, the human genetic and immunological determinants of COVID-19 pneumonia in the other 90% of children in this cohort remain unknown. With the CHGE, we recruited children hospitalized for COVID-19 pneumonia, including children with recessive inborn errors affecting type I IFNs (Zhang et al., 2022b). The rare children with autoimmune polyendocrine syndrome type I (APS-1), who harbor high titers of auto-Abs neutralizing type I IFNs from infancy onwards, are also known to be at high risk of life-threatening COVID-19 (Bastard et al., 2020, 2021c; Meisel et al., 2021; Valenzise et al., 2023). Furthermore, two Brazilian children hospitalized for severe COVID-19 were subsequently diagnosed with APS-1, following the identification of such auto-Abs (Schidlowski et al., 2022). We, therefore, tested the hypothesis that some of the unvaccinated children without APS-1 who suffered from COVID-19 pneumonia may also have harbored auto-Abs against type I IFNs before infection with SARS-CoV-2. We also assessed the prevalence of auto-Abs against type I IFNs in an uninfected pediatric population. We thus tested whether the main conclusions drawn with samples from adults, including both uninfected individuals and patients with various SARS-CoV-2 infections of various degrees of severity, also apply to children.

## Results

### Auto-Abs against type I IFNs in 19 of 183 children with COVID-19 pneumonia

**Figure S1.**
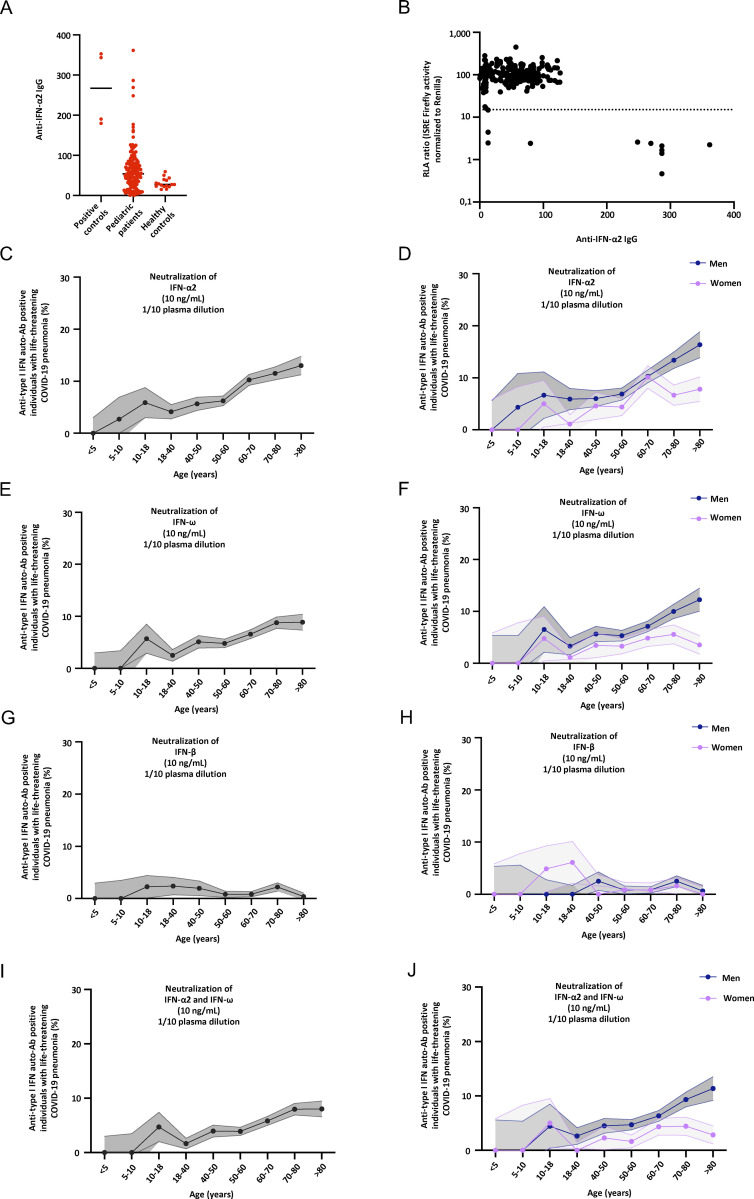
**Neutralizing auto-Abs against type I IFNs in children with life-threatening COVID-19. (A)** Gyros (high-throughput automated ELISA) results for auto-Abs against IFN-α2 for positive controls (*n* = 4), pediatric patients (*n* = 188), and healthy controls (*n* = 16). **(B)** Plot of anti–IFN-α2 auto-Ab IgG levels, as determined by Gyros, against their neutralization capacity at 10 ng/ml in the luciferase assay. For plasma from each patient, luciferase activity was normalized against the mean induction of control plasma tested on the same day in the luciferase assay. The horizontal dotted line indicates the threshold of neutralization, defined as the level of induction below 15% of the mean value for controls tested on the same day. **(C–J)** Proportion by age of pediatric and adult patients from the general population positive for neutralizing auto-Abs (in plasma 1:10) against (C) IFN-α2 at 10 ng/ml, for both sexes; (D) IFN-α2, at 10 ng/ml, for men or women; (E) IFN-ω, at 10 ng/ml, for both sexes; (F) IFN-ω, at 10 ng/ml, for men or women; (G) IFN-β, at 10 ng/ml, for both sexes; (H) IFN-β, at 10 ng/ml, for men or women; (I) IFN-α2 and IFN-ω, at 10 ng/ml, for both sexes; and (J) IFN-α2 and IFN-ω, at 10 ng/ml, for men or women.

**Figure 1.**
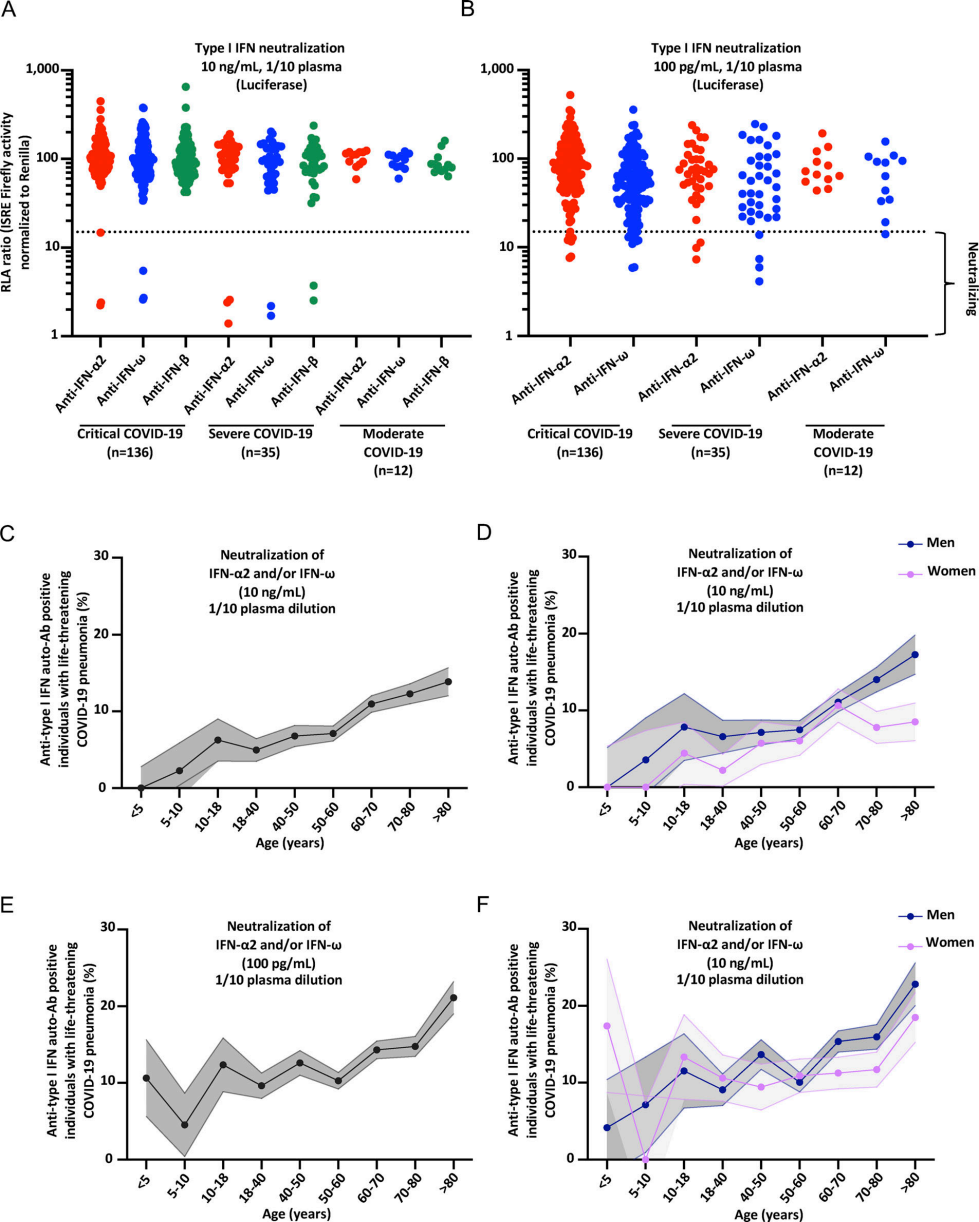
**Neutralizing auto-Abs against IFN-α2 and/or IFN-ω in children with life-threatening COVID-19. (A)** Results for the neutralization of 10 ng/ml IFN-α2, IFN-ω, or IFN-β in the presence of plasma (1/10 dilution) from pediatric patients with critical (*n* = 136), severe (*n* = 35), or moderate (*n* = 12) COVID-19 pneumonia. Relative luciferase activity is shown (IFN-stimulated response element [ISRE] dual luciferase activity, with normalization against *Renilla* luciferase activity) after stimulation with 10 ng/ml IFN-α2, IFN-ω, or IFN-β in the presence of plasma (1/10 dilution). RLA: relative luciferase activity. All samples were tested twice independently. **(B)** Neutralization of 100 pg/ml IFN-α2 or IFN-ω in the presence of plasma (1/10 dilution) from pediatric patients with critical (*n* = 136), severe (*n* = 35), or moderate (*n* = 12) COVID-19 pneumonia. All samples were tested twice independently. **(C–F)** The proportion by age of pediatric and adult patients with life-threatening COVID-19 pneumonia positive for neutralizing auto-Abs (in plasma 1/10) against (C) IFN-α2 and/or IFN-ω at 10 ng/ml for both sexes, (D) IFN-α2 and/or IFN-ω at 10 ng/ml for men or women, (E) IFN-α2 and/or IFN-ω at 100 pg/ml for both sexes, and (F) IFN-α2 and/or IFN-ω at 100 pg/ml for men or women.

We studied 183 previously healthy unvaccinated children hospitalized for COVID-19 pneumonia, including eight patients with one of the 15 known recessive IEI affecting type I IFNs (Zhang et al., 2022b) (see study flowchart). The patients were recruited via the CHGE and originated from nine countries (Brazil, France, Italy, Morocco, Saudi Arabia, Spain, Peru, Turkey, and Ukraine). The patients had a median age of 11 years and a mean age of 9 years (range: 0–17 years), and 50% were girls. As previously reported (Bastard et al., 2021a; Gervais et al., 2023), we used plasma or serum samples (diluted 1:10) from the patients for the assessment of anti-IFN-α2 IgG levels by Gyros (Fig. S1 A), and for assessments of neutralization activity against non-glycosylated IFN-α2 and IFN-ω at concentrations of 10 ng/ml and 100 pg/ml, and against glycosylated IFN-β at a concentration of 10 ng/ml (Fig. 1, A and B; and Table 1). The samples were obtained while the patients were hospitalized for COVID-19. The cohort studied here includes 53 of the 112 children previously reported in a study focusing on recessive IEI affecting type I IFNs (Zhang et al., 2022b). Only one of the eight children from our previously published cohort of 12 children with IEI affecting type I IFN immunity (Zhang et al., 2022b) tested had auto-Abs against type I IFNs. This patient had TLR7 deficiency and carried auto-Abs neutralizing 100 pg/ml IFN-ω. No plasma samples were available for the remaining four children from this cohort who were therefore not included in the cohort of 183 children studied here. Conversely, 130 of the children studied here were not included in the cohort investigated in the previous study (Zhang et al., 2022b). Our global cohort of 183 children consisted of 136 cases of critical disease requiring intensive care unit (ICU) hospitalization with high-flow oxygen (>6 L/min) supplementation or mechanical ventilation, 35 cases of severe COVID-19 pneumonia requiring <6 L/min of oxygen supplementation, and 12 with moderate infections that did not require oxygen supplementation. In this context, we identified 10 (5.5%) children with auto-Abs neutralizing IFN-α2: three (1.6%) with auto-Abs against IFN-α2 only, five (2.7%) with auto-Abs against IFN-α2 and IFN-ω, and two (1.1%) with auto-Abs against IFN-α2, IFN-ω, and IFN-β. In addition, nine children (4.9%) had auto-Abs neutralizing IFN-ω only. In total, 19 children (10.4%) had neutralizing auto-Abs against type I IFNs: 14 with critical, 4 with severe, and 1 with moderate COVID-19 pneumonia (Fig. 1, A and B; and Table 2). Moreover, plasma from 7 (3.8%) children contained auto-Abs that neutralized at least one IFN at a concentration of 10 ng/ml, whereas 12 (6.6%) children had auto-Abs neutralizing IFN only at a concentration of 100 pg/ml (Fig. 1, A and B). All patients with Gyros values over 200 had neutralizing auto-Abs (Fig. S1 B). Auto-Abs neutralizing IFN-α2, IFN-β, and/or IFN-ω were, thus, detected at the onset of COVID-19 pneumonia in 19 of the 183 unvaccinated children studied (10.4%): 10 (5.5%) with auto-Abs neutralizing IFN-α2 (three [1.6%] IFN-α2 only, five [2.7%] IFN-α2 and IFN-ω, and two [1.1%] IFN-α2, IFN-ω, and IFN-β), and nine (4.9%) with auto-Abs neutralizing IFN-ω only.

**Table 1.** Auto-Abs neutralized by the serum from the 19 patients

Patient	Anti-IFN-α2 auto-Abs (10 ng/ml)	Anti-IFN-β auto-Abs (10 ng/ml)	Anti-IFN-ω auto-Abs (10 ng/ml)	Anti-IFN-α2 auto-Abs (100 pg/ml)	Anti-IFN-ω auto-Abs (100 pg/ml)
P1	1	0	0	1	0
P2	1	1	1	1	1
P3	0	0	0	1	0
P4	0	0	0	0	1
P5	0	0	0	0	1
P6	0	0	0	0	1
P7	0	0	0	1	1
P8	1	0	1	1	1
P9	1	0	1	1	1
P10	0	0	0	1	0
P11	0	0	0	1	1
P12	1	0	0	1	1
P13	0	0	0	0	1
P14	1	1	1	1	1
P15	0	0	0	0	1
P16	0	0	1	0	1
P17	0	0	0	0	1
P18	0	0	0	0	1
P19	0	0	0	0	1

1: neutralizing. 0: non-neutralizing.

**Table 2.** Clinical and demographic information for the 19 pediatric patients with COVID-19 disease and auto-Abs neutralizing type I IFNs

Patient	Age	Sex	Country of origin	Country of residence	Classification
P1	10	M	Turkey	Turkey	Critical
P2	14	F	France	France	Severe
P3	12	F	Turkey	Turkey	Critical
P4	17	F	Turkey	Turkey	Critical
P5	17	M	Turkey	Turkey	Moderate
P6	3	M	Turkey	Turkey	MIS-C + critical
P7	15	M	Turkey	Turkey	Critical
P8	18	M	France	France	Critical
P9	18	M	Turkey	Turkey	Critical
P10	0.25	F	Turkey	Turkey	Critical
P11	0.5	F	France	France	Critical
P12	14	M	Turkey	Turkey	Severe
P13	7.3	M	Turkey	Turkey	Severe
P14	11	F	Pakistan	Italy	Severe
P15	4	F	Turkey	Turkey	Critical
P16	13	M	Turkey	Turkey	Critical
P17	15	F	Turkey	Turkey	Critical
P18	16	F	Turkey	Turkey	Critical
P19	2	F	Spain	Spain	Critical

F: female; M: male.


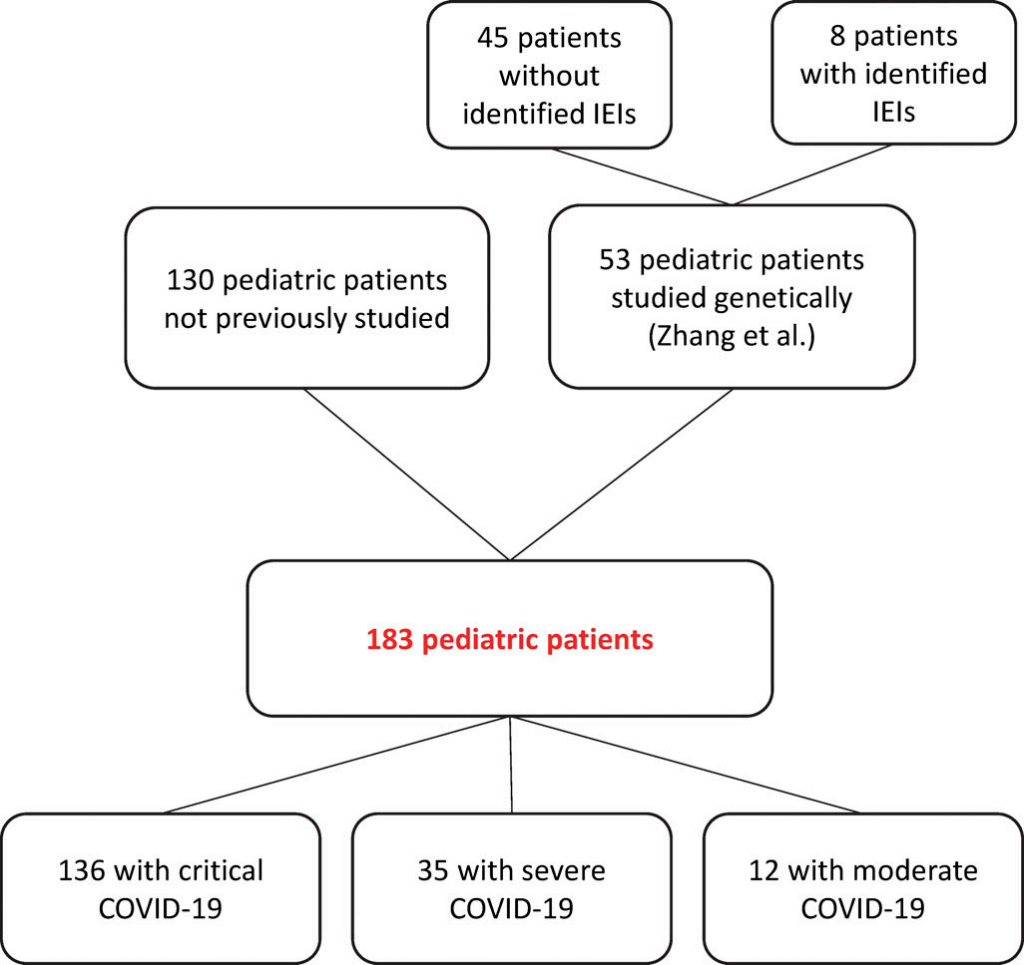


### Demographic and clinical features of the 19 patients with auto-Abs against type I IFNs

**Figure S2.**
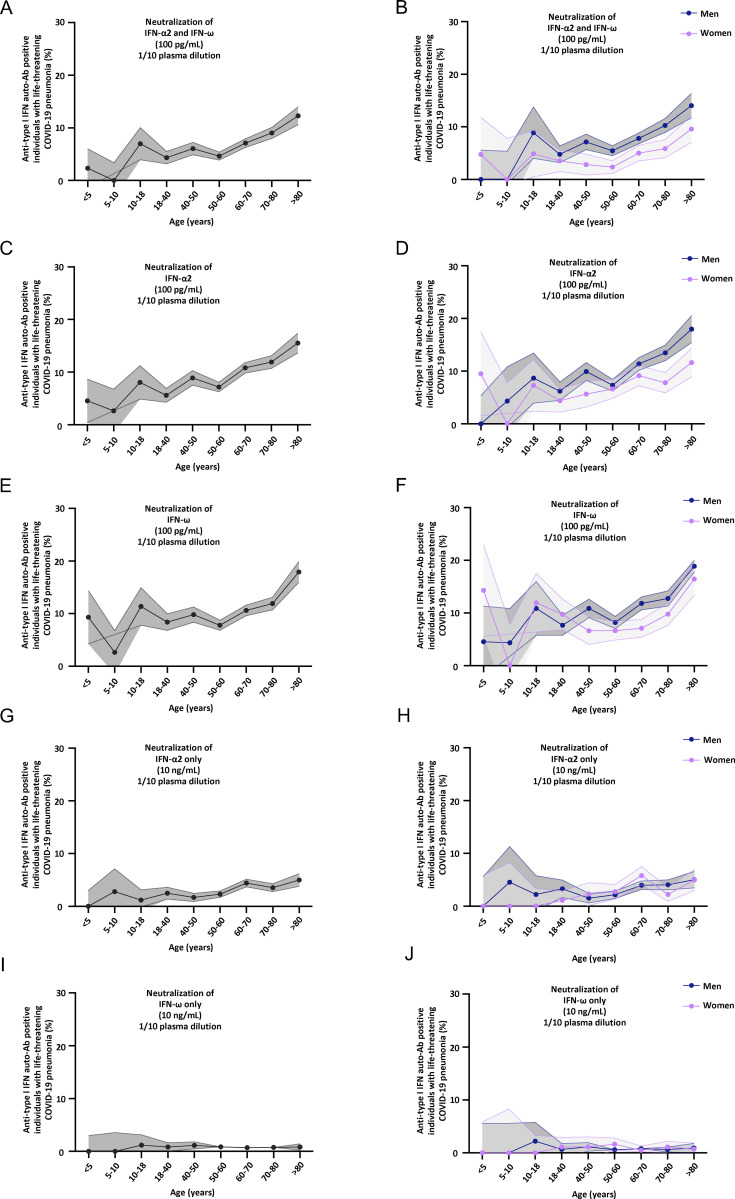
**Neutralizing auto-Abs against IFN-α2 and/or IFN-ω in children and adults with life-threatening COVID-19. (A–J)** Proportion by age of pediatric and adult patients from the general population positive for neutralizing auto-Abs (in plasma 1:10) against (A) IFN-α2 and IFN-ω, at 100 pg/ml, for both sexes; (B) IFN-α2 and IFN-ω, at 100 pg/ml, for men or women; (C) IFN-α2, at 100 pg/ml, for both sexes; (D) IFN-α2, at 100 pg/ml, for men or women; (E) IFN-ω, at 100 pg/ml, for both sexes; (F) IFN-ω, at 100 pg/ml, for men or women; (G) IFN-α2 only, at 10 ng/ml, for both sexes; (H) IFN-α2 only, at 10 ng/ml, for men or women; (I) IFN-ω only, at 10 ng/ml, for both sexes; and (J) IFN-ω only, at 10 ng/ml, for men or women.

The 19 children with COVID-19 pneumonia and auto-Abs neutralizing type I IFNs comprised 10 girls and 9 boys, aged 3 mo to 18 years (mean: 11 years) (Fig. 1, C–F, Fig. S1, C–J, Fig. S2, A–J; and Table 3). They originated from four different countries (France, Pakistan but living in Italy, Spain, and Turkey), with a particularly large proportion of patients from Turkey (79%). Turkish children did not account for a disproportionate number of the individuals with auto-Abs against type I IFNs (79% of Turkish children with antibodies (Abs) versus 66% without; P = 0.31; Fisher’s exact test). None of these individuals had previously suffered from other severe viral infections known to be associated with these auto-Abs, such as live attenuated yellow fever viral vaccine disease (Bastard et al., 2021b), West Nile virus encephalitis (Gervais et al., 2023), critical influenza pneumonia (Zhang et al., 2022c), critical MERS pneumonia (Alotaibi et al., 2023), or severe zoster infection (Busnadiego et al., 2022; Mathian et al., 2022; Mogensen et al., 1981; Pozzetto et al., 1984; Walter et al., 2015). The two children under the age of 6 mo may have received the auto-Abs via materno-fetal transmission. APS-1 was excluded clinically (no other clinical features of autoimmunity) in all 19 patients and genetically in all 12 for whom DNA samples were available. Only 1 of the 12 children for whom DNA was available carried any of the known IEI-affecting type I IFNs. This patient produced auto-Abs neutralizing the lower concentration of IFN-ω and had X-linked TLR7 deficiency (Asano et al., 2021). All the children were hospitalized for pneumonia following SARS-CoV-2 infection. Among the 19 children with auto-Abs neutralizing type I IFNs, 1 (5%) with auto-Abs neutralizing IFN-ω only (at a concentration of 100 pg/ml) had moderate COVID-19 pneumonia, 4 children (21%) were hospitalized for severe COVID-19 pneumonia, and 14 children (74%) had critical disease (Fig 1, A and B; and Table 3). One of the children with critical disease had cardiological, neurological, cutaneous, and gastrointestinal manifestations of the multisystem inflammatory syndrome in children (MIS-C) (Lee et al., 2023; Sancho-Shimizu et al., 2021). All the patients survived, and all except the patients with additional manifestations had positive SARS-CoV-2 RT-PCR results on samples from the respiratory tract. The patient with additional manifestations had critical pneumonia and a subsequent positive serological test demonstrated infection. Overall, these findings suggest that auto-Abs against type I IFNs can underlie life-threatening COVID-19 pneumonia in a significant proportion of previously healthy unvaccinated children.

**Table 3.** Numbers of cases, proportion, and OR for COVID-19 pneumonia in pediatric patients

Auto-Abs (dose)	Number of patients positive	Proportion of patients testing positive (%)	OR [95% CI] for COVID-19 pneumonia	P value
Anti-IFN-α2 (10 ng/ml)	6	3.4	57 [12–560]	3 × 10^−07^
Anti-IFN-β (10 ng/ml)	2	1.1	23 [3–255]	4 × 10^−03^
Anti-IFN-ω (10 ng/ml)	5	2.8	9 [3–29]	5 × 10^−04^
Anti-IFN-α2 (100 pg/ml)	10	5.5	30 [10–103]	1 × 10^−9^
Anti-IFN-ω (100 pg/ml)	16	8.8	5 [2–8]	7 × 10^−06^
Anti-IFN-α2 and/or anti-IFN-ω (10 ng/ml)	7	3.9	11 [4–32]	2 × 10^−05^
Anti-IFN-α2 and/or anti-IFN-β, and/or anti-IFN-ω (10 ng/ml)	7	3.8	11 [4–29]	3 × 10^−05^
Anti-IFN-α2 and anti-IFN-ω (10 ng/ml)	4	2.4	112 [12–14,991]	9 × 10^−04^
Anti-IFN-α2, anti-IFN-ω, and anti-IFN-β (10 ng/ml)	2	1.1	75 [6–10,328]	9 × 10^−04^
Anti-IFN-α2 only (10 ng/ml)	2	1.1	23 [3–263]	4 × 10^−03^
Anti-IFN-ω only (10 ng/ml)	1	0.6	3 [0.3–12]	3 × 10^−01^
Anti-IFN-β only (10 ng/ml)	0	0.00	4 [0.03–57]	5 × 10^−01^
Anti-IFN-α2 and/or anti-IFN-ω (100 pg/ml)	19	10.4	5 [3–9]	1 × 10^−07^
Anti-IFN-α2 and anti-IFN-ω (100 pg/ml)	7	3.9	26 [7–106]	7 × 10^−07^

### Auto-Abs neutralize all 12 IFN-α subtypes

**Figure 2.**
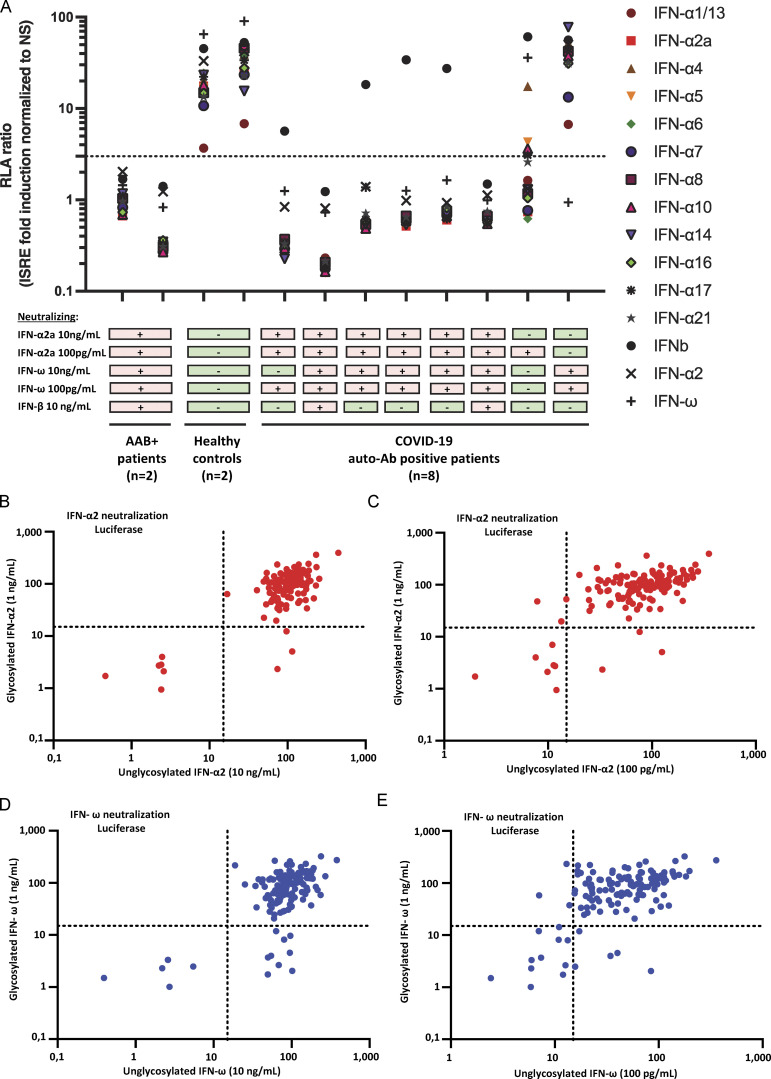
**Auto-Abs neutralize all 12 IFN-α subtypes and the glycosylated IFNs. (A)** Results for the neutralization of 12 individual IFN-α subtypes, IFN-ω or IFN-β, from children with auto-Abs (*n* = 8), healthy controls (*n* = 2), or auto-Ab positive patients (*n* = 2). Relative luciferase activity is shown (ISRE dual luciferase activity, with normalization against *Renilla* luciferase activity, and to the non-stimulated [NS] condition) after stimulation with the various type I IFNs at a concentration of 1 ng/ml in the presence of plasma (1/10 dilution). All samples were tested once. RLA: relative luciferase activity. **(B–E)** Plots representing the neutralization results for glycosylated (at 1 ng/ml) or unglycosylated (at 10 ng/ml or 100 pg/ml) forms of IFN-α2 and IFN-ω. The dots in the lower right part of the plot indicate neutralization of the glycosylated form of the IFN but not of the unglycosylated form, whereas the dots in the upper right part of the plot indicate neutralization of the unglycosylated form of the IFN but not of the glycosylated form. The dots in the lower left part of the plot indicate the neutralization of either form of the IFNs, whereas those in the upper right part of the plot indicate an absence of neutralization for both forms; (B) unglycosylated form of IFN-α2 at 10 ng/ml; (C) unglycosylated form of IFN-α2 at 100 pg/ml; (D) unglycosylated form of IFN-ω at 10 ng/ml; and (E) unglycosylated form of IFN-ω at 100 pg/ml.

We assessed the neutralization of the 12 individual IFN-α subtypes at the intermediate concentration of 1 ng/ml. There are 13 *IFNA* loci, but only 12 IFN-α proteins, as the products of *IFNA1* and *IFNA13* are identical (Moreau et al., 2023). Interestingly, for all patients with auto-Abs neutralizing 10 ng/ml IFN-α2, all 12 IFN-α subtypes were neutralized (Fig. 2 A). None of the patients with auto-Abs against IFN-ω but without detectable auto-Abs against IFN-α2 displayed neutralization of any of the 12 IFN-α subtypes. However, the patient tested for whom IFN-α2 neutralization was observed at 100 pg/ml, but not 10 ng/ml, also displayed neutralization at a concentration of 1 ng/ml, and neutralization of most of the other IFN-α subtypes (no neutralization of IFN-α4/5/10). We did not assess the neutralization of these IFNs at 100 pg/ml. These findings are consistent with the high degree of similarity between the 12 IFN-α subtypes (Manry et al., 2011) and the presence of a B cell epitope recognized by the auto-Abs in a conserved region of these IFNs (Meyer et al., 2016). It also suggests that patients with auto-Abs neutralizing all IFN-α subtypes might be at higher risk of severe viral disease.

### Auto-Abs neutralize glycosylated IFNs

**Figure S3.**
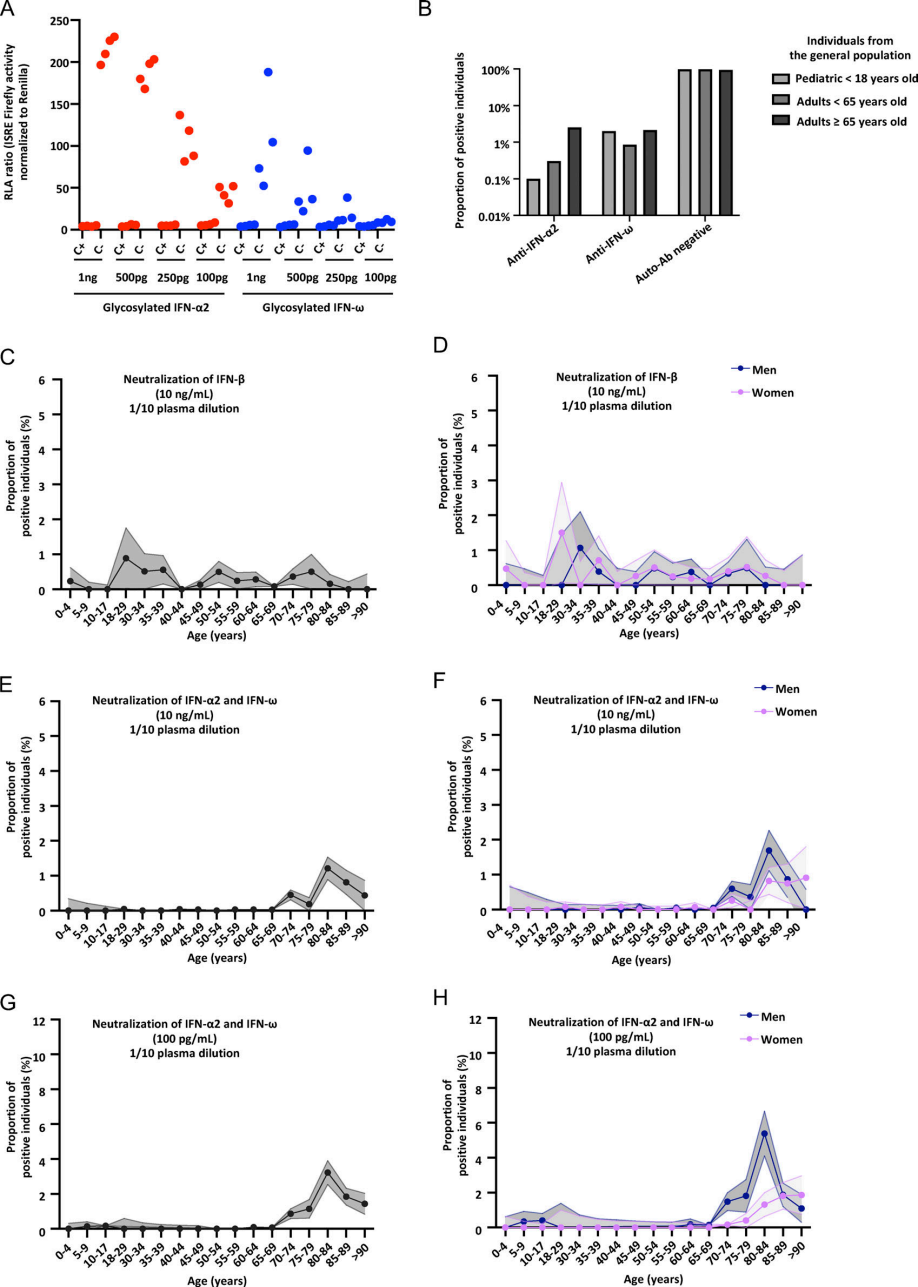
**Neutralizing auto-Abs against glycosylated type I IFNs, and proportion of children and adults from the general population with neutralizing auto-Abs against type I IFNs. (A)** Results for the neutralization of various doses of the glycosylated form of IFN-α2 or IFN-ω in the presence of plasma (1/10 dilution) from children with (C−) and without (C+) auto-Abs neutralizing type I IFNs. Relative luciferase activity is shown (ISRE dual luciferase activity, with normalization against *Renilla* luciferase activity) after stimulation with 10 ng/ml IFN-α2 or IFN-ω in the presence of plasma (1/10 dilution). RLA: relative luciferase activity. **(B–H)** Proportion of children and adults from the general population with neutralizing auto-Abs against type I IFNs. **(B)** Prevalence of auto-Abs neutralizing type I IFNs, by type of IFN neutralized. **(C–H)** Proportion, by age, of pediatric and adult individuals from the general population positive for neutralizing auto-Abs (in plasma diluted 1:10) against (C) IFN-β, at 10 ng/ml, for both sexes; (D) IFN-β, at 10 ng/ml, for men or women; (E) IFN-α2 and IFN-ω, at 10 ng/ml, for both sexes; (F) IFN-α2 and IFN-ω, at 10 ng/ml, for men or women; (G) IFN-α2 and IFN-ω, at 100 pg/ml, for both sexes; and (H) IFN-α2 and IFN-ω, at 100 pg/ml, for men or women.

In our previous studies, we tested only unglycosylated IFN-α2a, IFN-α14, and IFN-ω produced in cells of the bacterium *Escherichia coli* and glycosylated IFN-β produced by mammalian CHO cells (Adolf et al., 1991; Nyman et al., 1998; Runkel et al., 1998). Here, we considered four human type I IFNs (IFN-α2a/b, IFN-α14, IFN-ω, and IFN-β) normally produced and secreted as glycosylated proteins. IFN-α2b is produced as an O-glycosylated form, whereas IFN-α2a is present in two forms, one fully and the other partially O-glycosylated. By contrast, IFN-α14, IFN-ω, and IFN-β are produced as N-glycosylated forms. We, therefore, investigated the effects of glycosylation on the recognition of these proteins by auto-Abs by determining whether auto-Abs recognized glycosylated but not unglycosylated IFNs or vice versa. We, therefore, tested the neutralization of glycosylated forms of IFN-α2b and IFN-ω produced in mammalian cells. We first determined the optimal experimental set-up. We found that the optimal concentration for testing was 1 ng/ml for glycosylated type I IFN (Fig. S3 A). We tested 183 children, including 19 with auto-Abs against type I IFNs. Most of the 19 patients with auto-Abs neutralizing unglycosylated IFN-α2 or IFN-ω also displayed neutralization of the glycosylated forms. However, three patients had auto-Abs that neutralized the unglycosylated but not the glycosylated form of IFN-ω, and three had auto-Abs neutralizing the unglycosylated but not the glycosylated form of IFN-α2, at a concentration of 1 ng/ml (Fig. 2, B–E). Interestingly, we also found two patients with auto-Abs neutralizing the glycosylated forms of both IFN-ω and IFN-α2b but not the unglycosylated form of either cytokine, and one patient with auto-Abs neutralizing the glycosylated form of IFN-α2b but not the unglycosylated form. Another two patients had auto-Abs neutralizing the glycosylated form of IFN-ω but not the unglycosylated form. We did not test the unglycosylated form of IFN-β. Serum from the six patients with auto-Abs neutralizing 10 ng/ml unglycosylated IFN-α2 also neutralized all 12 IFN-α subtypes and glycosylated IFN-α2 at a concentration of 1 ng/ml. Interestingly, 2 of the 183 patients tested had auto-Abs neutralizing the glycosylated but not the unglycosylated form of IFN-α2b or IFN-ω, whereas 3 had auto-Abs neutralizing the unglycosylated forms of both cytokines but not the glycosylated forms. These findings suggest that it may be useful to assess the neutralization of glycosylated forms of IFN-α2a and IFN-ω as a means of identifying previously unrecognized patients.

### Auto-Abs neutralizing type I IFNs are rare in children from the general population

**Figure 3.**
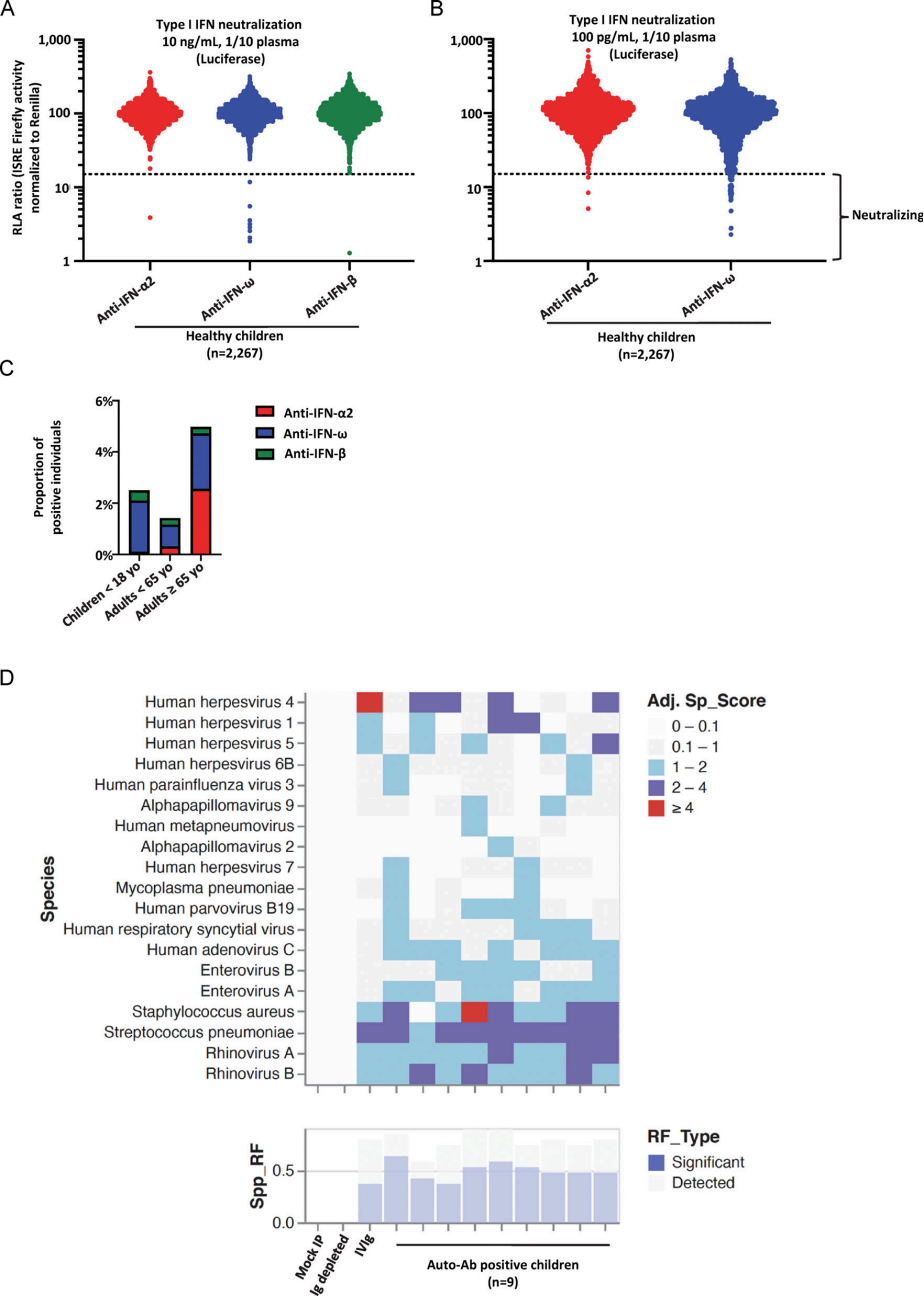
**Neutralizing auto-Abs against IFN-α2 and/or IFN-ω in children from the general population and their serological evaluation. (A)** Results for the neutralization of 10 ng/ml IFN-α2, IFN-ω, or IFN-β in the presence of plasma (1/10 dilution) from children from the general population (*n* = 2,267). Relative luciferase activity is shown (ISRE dual luciferase activity, with normalization against *Renilla* luciferase activity). All samples were tested once. RLA: relative luciferase activity. **(B)** Neutralization of 100 pg/ml IFN-α2 or IFN-ω in the presence of plasma (1/10 dilution) from children from the general population (*n* = 2,267). **(C)** Prevalence of auto-Abs neutralizing type I IFNs, distributed by age, in individuals from the general population. **(D)** Serological evaluation of auto-Ab-positive children: Virscan results for children (*n* = 9) with auto-Abs against type I IFNs.

**Figure 4.**
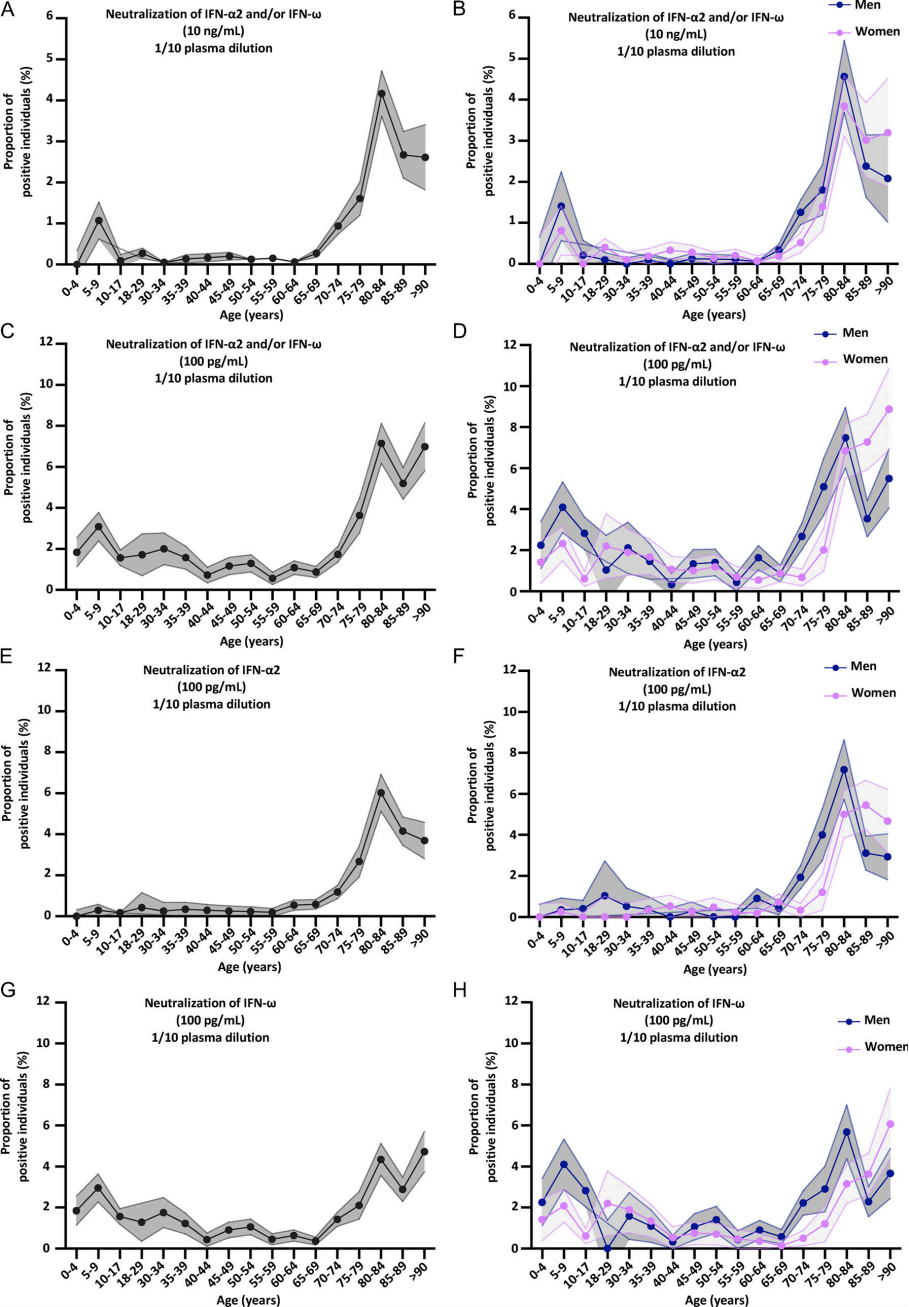
**Neutralizing auto-Abs against IFN-α2 and/or IFN-ω in the pediatric and adult general population. (A–H)** Proportion by age of pediatric and adult individuals from the general population positive for neutralizing auto-Abs (in plasma 1/10) against (A) IFN-α2 and/or IFN-ω, at 10 ng/ml, for both sexes; (B) IFN-α2 and/or IFN-ω, at 10 ng/ml, for men or women; (C) IFN-α2 and/or IFN-ω, at 100 pg/ml, for both sexes; (D) IFN-α2 and/or IFN-ω, at 100 pg/ml, for men or women; (E) IFN-α2, at 100 pg/ml, for both sexes; (F) IFN-α2, at 100 pg/ml, for men or women; (G) IFN-ω, at 100 pg/ml, for both sexes; and (H) IFN-ω, at 100 pg/ml, for men or women.

**Figure S4.**
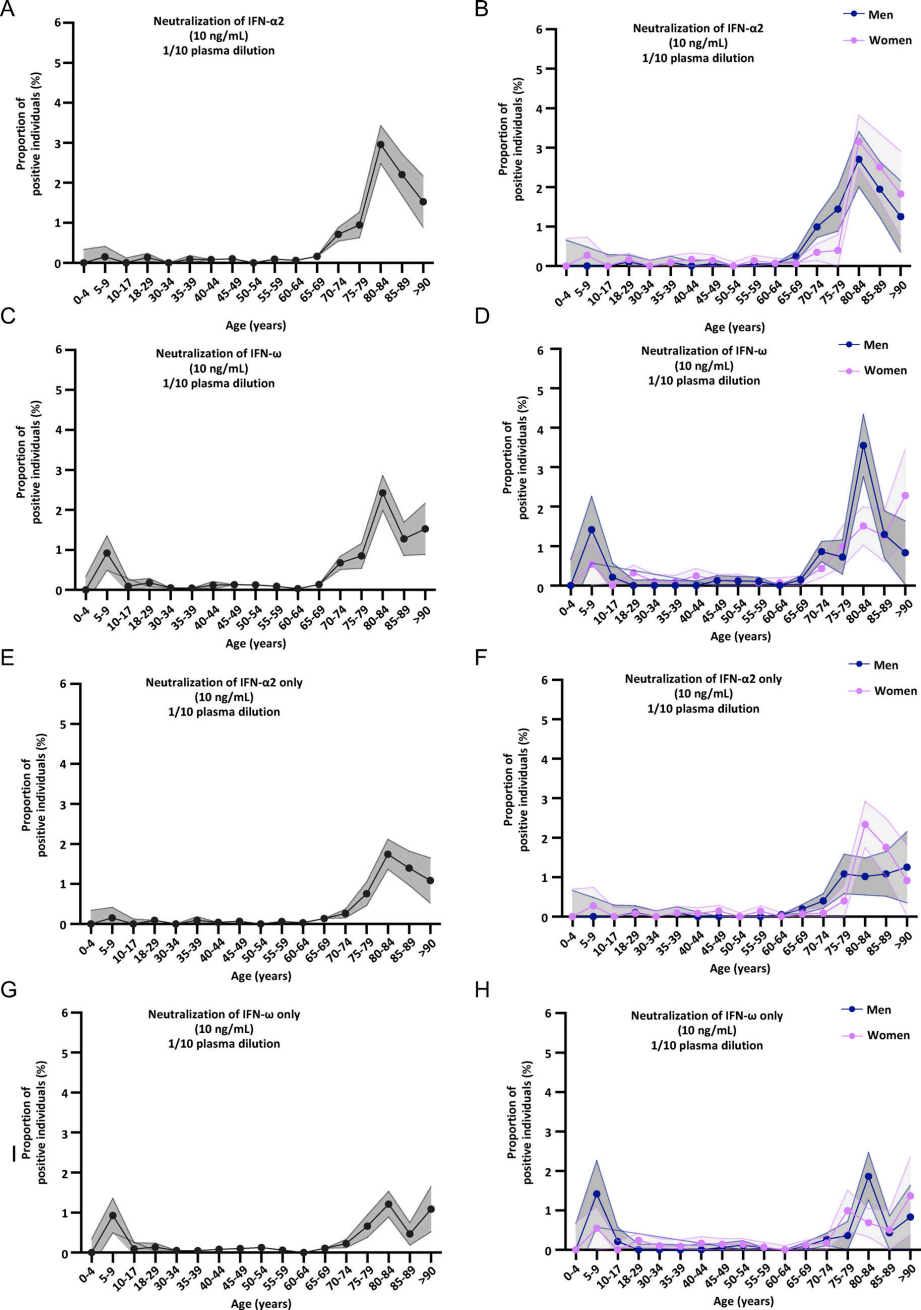
**Neutralizing auto-Abs against IFN-α2 or IFN-ω in children from the general population. (A–H)** Proportion, by age, of pediatric and adult individuals from the general population positive for neutralizing auto-Abs (in plasma diluted 1:10) against (A) IFN-α2, at 10 ng/ml, for both sexes; (B) IFN-α2, at 10 ng/ml, for men or women; (C) IFN-ω, at 10 ng/ml, for both sexes; (D) IFN-ω, at 10 ng/ml, for men or women; (E) IFN-α2 only, at 10 ng/ml, for both sexes; (F) IFN-α2 only, at 10 ng/ml, for men or women; (G) IFN-ω only, at 10 ng/ml, for both sexes; and (H) IFN-ω only, at 10 ng/ml, for men or women.

**Figure S5.**
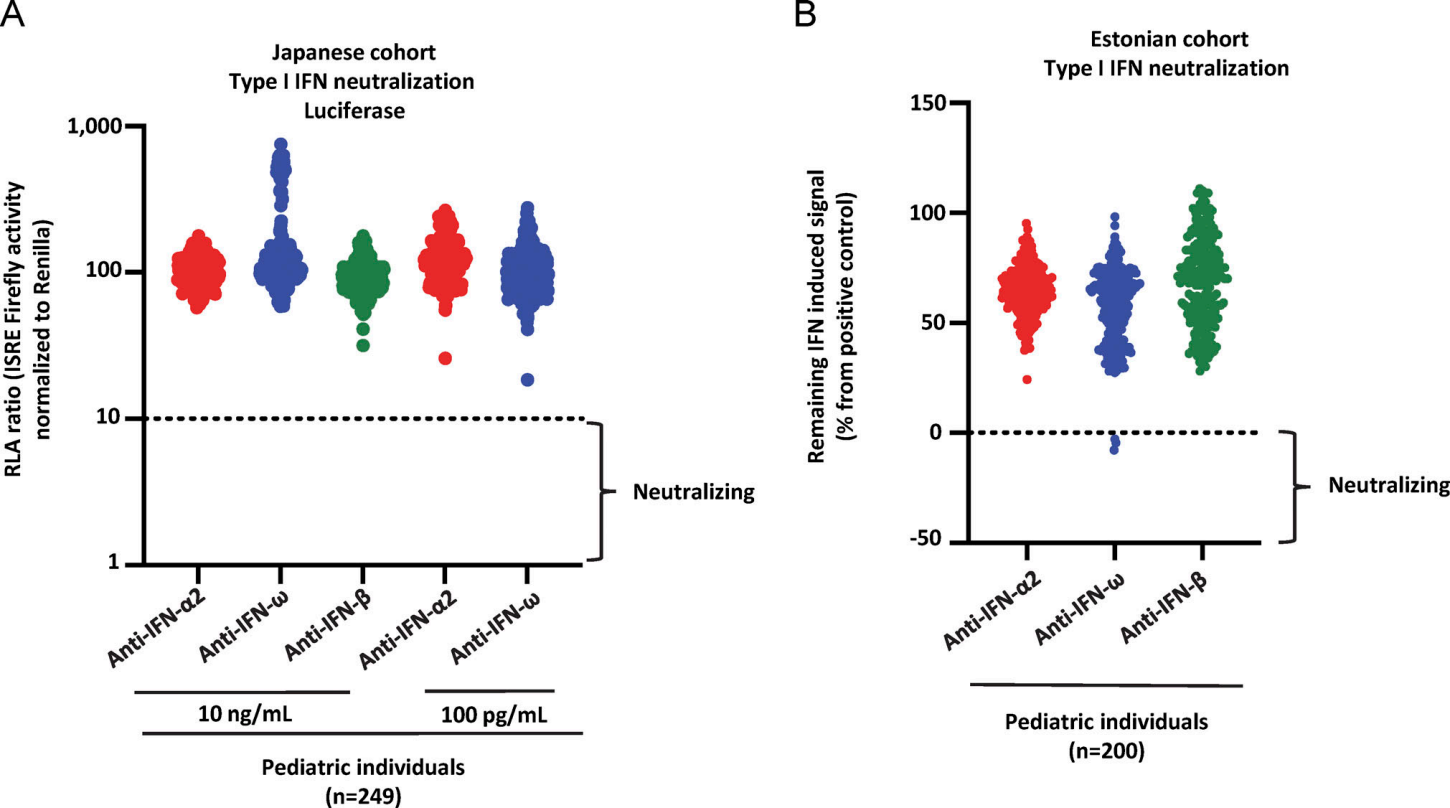
**Neutralizing auto-Abs against IFN-α2 and/or IFN-ω in children from the general population in Estonia and Japan. (A)** Results for the neutralization of 10 ng/ml IFN-α2, IFN-ω, or IFN-β or 100 pg/ml IFN-α2 or IFN-ω in the presence of plasma (1/10 dilution) from children from Japan (*n* = 249). **(B)** Results for the neutralization of 10 ng/ml or 100 pg/ml IFN-α2, IFN-ω, or IFN-β in the presence of plasma (1/10 dilution) from children from Estonia (*n* = 200). **(A and B)** Relative luciferase activity is shown (ISRE dual luciferase activity, with normalization against *Renilla* luciferase activity) after stimulation with IFN-α2, IFN-ω, or IFN-β in the presence of plasma (1/10 dilution). RLA: relative luciferase activity.

We previously tested large adult cohorts, comprising a total of 39,198 individuals, to assess the prevalence of auto-Abs against type I IFNs in the uninfected general population (Bastard et al., 2020, 2021a; van der Wijst et al., 2021). The prevalence of auto-Abs neutralizing 10 ng/ml (or 100 pg/ml) IFN-α2 or IFN-ω was found to increase significantly with age, with the detection of such Abs in 0.17% (1.1%) of individuals under and >1.4% (4.4%) of those over the age of 70 years, making a major contribution to the higher risk of life-threatening COVID-19 in the elderly population (Manry et al., 2022). The prevalence of auto-Abs against IFN-β was lower and remained stable across age groups (0.26%) (Bastard et al., 2020). Interestingly, auto-Abs neutralizing IFN-α2 (at the lower concentration of 100 pg/ml), regardless of the presence or absence of auto-Abs neutralizing IFN-ω, were found in 0.3% of individuals under the age of 70 years, whereas those neutralizing 100 pg/ml IFN-ω were found in 0.9% of this population (Table 4). Strikingly, the prevalence of auto-Abs neutralizing IFN-α2 increased eightfold after the age of 65 years, whereas the prevalence of auto-Abs neutralizing IFN-ω increases only 2.5-fold (Fig. 3 C, Fig. 4, A–H; Fig. S3, B–H; Fig. S4, A–H; and Table 4). In men, the increase in the prevalence of auto-Abs against IFN-α2 was even greater, >10-fold after the age of 65 years. We therefore assessed the prevalence of these auto-Abs in 2,267 children from the general population with samples collected before the pandemic and, therefore, before any possibility of infection with SARS-CoV-2. Samples were collected in Belgium (*n* = 126), Canada (*n* = 161), Estonia (*n* = 288), Spain (*n* = 1,685), and Pakistan (*n* = 7). The children studied were aged 0–18 years (median and mean ages: 10 and 9 years, respectively), with an equal distribution between the sexes (56% were girls). Interestingly, in children, auto-Abs neutralizing IFN-α2 were exceedingly rare. Indeed, only one child (0.04%) had auto-Abs neutralizing IFN-α2 at 10 ng/ml and only three children (0.1%) had auto-Abs neutralizing this cytokine at a concentration of 100 pg/ml. The auto-Abs of these three children also neutralized IFN-ω. By contrast, auto-Abs neutralizing IFN-ω alone were found in a much higher proportion of uninfected children. Indeed, eight children (0.35%) had auto-Abs neutralizing IFN-ω at 10 ng/ml, whereas an additional 38 (1.7%) had auto-Abs neutralizing IFN-ω at 100 pg/ml (Fig. 3, A and B). We also identified one girl, aged 1.5 years, with auto-Abs neutralizing only glycosylated IFN-β at a concentration of 10 ng/ml (0.04%) (Fig. 3 A). Finally, we tested two additional independent cohorts of healthy children. None of the individuals of a cohort of 249 healthy children aged 0–18 years (median: 9 years) from Japan tested positive (Fig. S5 A). These pediatric controls included 34 individuals who had had mild COVID infection and did not harbor auto-Abs against type I IFNs. A cohort of 200 healthy children from Estonia (all aged 8–9 years) included only three individuals with auto-Abs neutralizing IFN-ω at a concentration of 100 pg/ml (1.5%) (Fig. S5 B). Overall, 0.17% of uninfected children from our cohort had auto-Abs neutralizing IFN-α2 (4 of 2,267, including 3 at low and 1 at high concentration), whereas 0.04% had auto-Abs neutralizing glycosylated IFN-β (1 of 2,267, at 10 ng/ml) and 2% had auto-Abs neutralizing IFN-ω only (46 of 2,267, including 38 at low and 8 at high concentration). The neutralization of two IFNs simultaneously was exceedingly rare and restricted to IFN-α2 and IFN-ω at the lower concentration in three patients (0.1%).

**Table 4.** Prevalence of auto-Abs against type I IFNs in the general population

Type I IFN auto-Ab (in plasma 1/10)	Age	Proportion of individuals from the general population with neutralizing auto-Abs (%)
Anti-IFN-α2 (100 pg/ml)	Children <18 years	0.2%
	Adults <65 years	0.3%
	Adults ≥65 years	2.6%
Anti-IFN-ω (100 pg/ml)	Children <18 years	2%
	Adults <65 years	0.9%
	Adults ≥65 years	2.2%

### Characteristics of children from the general population with auto-Abs against type I IFNs

We found that 2,267 children from the general population tested included 4 with auto-Abs neutralizing IFN-α2 (0.2%) and 45 with auto-Abs neutralizing IFN-ω (2%). Three (6%) of these children had auto-Abs neutralizing both IFN-α2 and IFN-ω. These three children were aged 8, 11, and 13 years, and all three were boys. The individual with auto-Abs neutralizing IFN-α2 only was a 9-year-old girl. Finally, the median age of the 42 children with auto-Abs neutralizing IFN-ω only was 8 years, and 31 (67%) of these children were boys. We also tested a cohort of 145 samples from children hospitalized for bacterial infections. Only one of these patients (0.7%) harbored auto-Abs against type I IFNs (against IFN-ω only) with neutralizing activity against a concentration of 100 pg/ml. None of these children had auto-Abs neutralizing IFN-α2 or IFN-β. None of the children with auto-Abs tested had any remarkable medical antecedents, despite having been infected with many viruses, as shown by Virscan analyses on nine of the positive children (Fig. 3 D). However, it should be noted that infections with influenza viruses or common coronaviruses were not investigated with Virscan. These findings probably attest to the higher tonic type I IFN activity in children than in adults (Loske et al., 2021; Pierangeli et al., 2022; Pierce et al., 2021). Overall, we found that auto-Abs neutralizing IFN-α2 were very rare in children from the general population. By contrast, auto-Abs neutralizing IFN-ω only, at the lower concentration, were less rare (2%) and mostly found in boys, at a rate slightly higher than that for young adults under the age of 40 years (45/2,267 [2%] in children versus 17/1,251 [1.4%] in adults between 18 and 40 years old, P = 0.28).

### Risk of life-threatening COVID-19 in children with auto-Abs against type I IFNs

**Figure 5.**
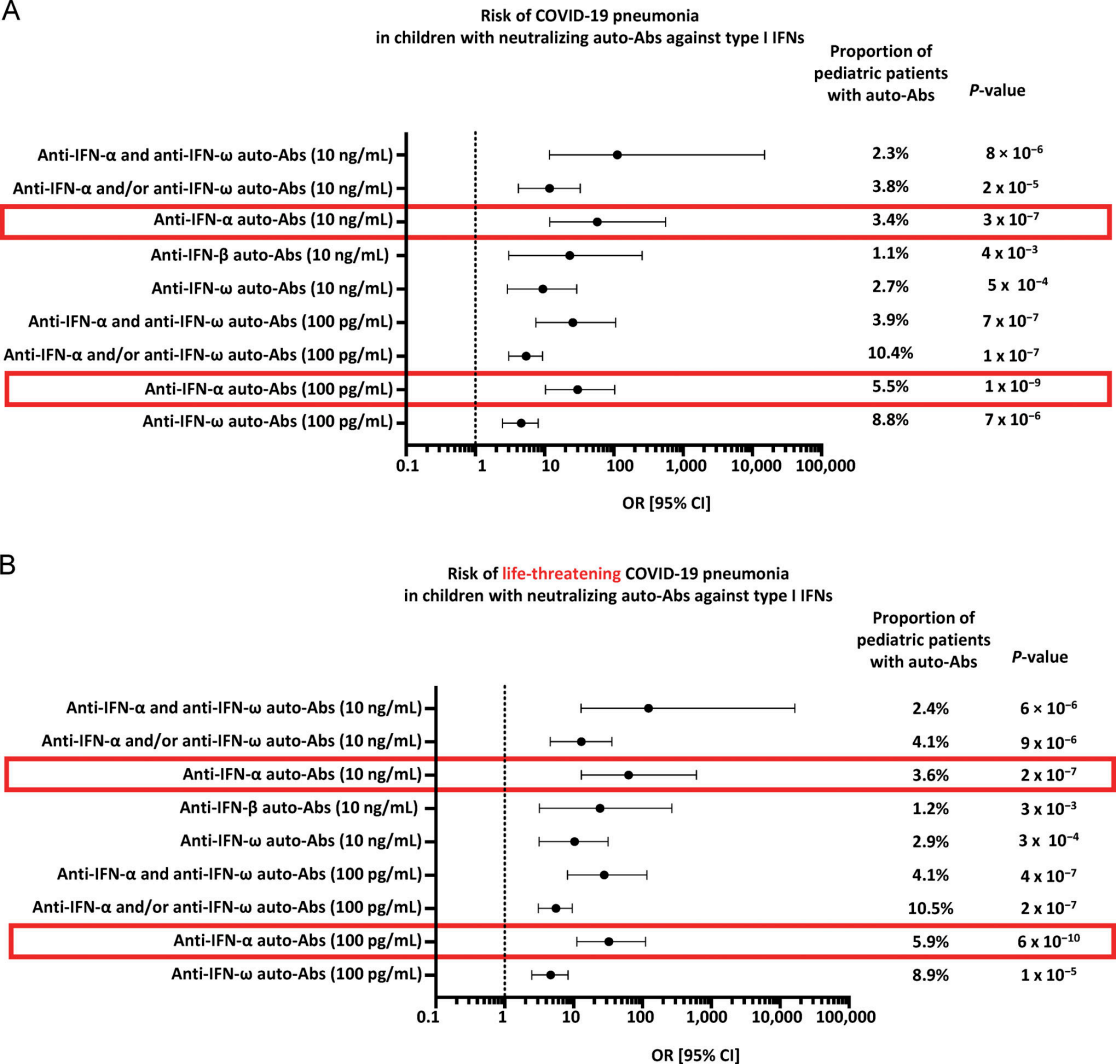
**OR for COVID-19 pneumonia. (A)** Bar plot of the calculated OR for COVID-19 pneumonia in children with auto-Abs against type I IFNs. Adjusted ORs and 95% CI were computed by penalized profile likelihood. ORs and P values were estimated by means of Firth’s bias-corrected logistic regression. **(B)** Bar plot of the calculated OR for life-threatening pneumonia in children with auto-Abs against type I IFNs. Adjusted ORs and 95% CI were computed by penalized profile likelihood. ORs and P values were estimated by means of Firth’s bias-corrected logistic regression.

We then assessed the risk of COVID-19 pneumonia (hospitalization for hypoxemic pneumonia, including severe or critical pneumonia) in children carrying auto-Abs capable of neutralizing different concentrations and combinations of type I IFNs, relative to uninfected children from the general population, as previously reported for COVID-19 and influenza in adults (Bastard et al., 2021a; Manry et al., 2022). All types of auto-Ab combinations were highly significant risk factors when patients with severe or critical COVID-19 pneumonia were compared with the general population (Fig. 5 and Table 2). The strongest association with severe or critical pneumonia was that for children with auto-Abs neutralizing both IFN-α2 and IFN-ω at a concentration of 10 ng/ml (OR [95% CI] = 122.8 [12.8–16,364.8], P = 6 × 10^−6^; OR, odds ratio; CI, confidence interval). Auto-Abs neutralizing IFN-α2 and IFN-ω at a lower concentration of 100 pg/ml were also highly significant risk factors (OR [95% CI] = 27.9 [8.2–116.5], P = 4 × 10^−7^), whereas auto-Abs neutralizing IFN-α2 or IFN-ω were weaker risk factors (OR [95% CI] = 5.5 [3.1–9.6] at 100 pg/ml and OR [95% CI] = 12.9 [4.6–35.9] at 10 ng/ml), with the OR for auto-Abs neutralizing high concentrations of IFN-α2 or IFN-ω significantly higher than that for auto-Abs neutralizing low concentrations of IFN-α2 or IFN-ω (P = 0.006). The risk of life-threatening COVID-19 pneumonia did not differ significantly between children with auto-Abs neutralizing only IFN-α2 at 100 pg/ml (OR [95% CI] = 67.6 [5.7–9,196.6]) and those with auto-Abs neutralizing IFN-α2 and IFN- ω (OR [95% CI] = 27.9 [8.2–116.5], P = 0.5), but the risk for children with auto-Abs neutralizing only IFN-ω at a concentration of 100 pg/ml was significantly lower (OR [95% CI] = 2.6 [1.2–5.3], P = 0.006). Overall, auto-Abs neutralizing IFN-α2 were significantly stronger risk factors than auto-Abs neutralizing only IFN-ω, both at 10 ng/ml (OR [95% CI] = 62.9 [12.9–610.3] for auto-Abs neutralizing IFN-α2 versus 2.8 [0.3–13.2] for IFN-ω only, P = 0.0003) and 100 pg/ml (OR [95% CI] = 32.5 [11.2–111.2] for auto-Abs neutralizing IFN-α2 versus 2.6 [1.2–5.3] for IFN-ω only, P = 0.03), consistent with the higher prevalence of auto-Abs neutralizing IFN-ω in the general population (Table 2). Overall, these findings indicate that auto-Abs against type I IFNs can be found in previously healthy children in whom they are a major risk factor for hypoxemic COVID-19 pneumonia, particularly if they neutralize IFN-α2. Indeed, auto-Abs against IFN-α confer the highest risk of life-threatening COVID-19. Interestingly, the ORs for critical COVID-19 were also higher for adults with auto-Abs neutralizing IFN-α than for those neutralizing IFN-ω, in comparison to adults with critical COVID-19 and asymptomatic infected adult controls (P = 0.008; Table 5). Moreover, the risk of hypoxemic pneumonia increases with the concentration of type I IFN neutralized in children and adults (Table 2 for children, Table 5 for adults).

**Table 5.** Increase in the risk of critical COVID-19 in adult patients with auto-Abs against IFN-α (neutralizing 10 ng/ml) versus IFN-ω (neutralizing at 10 ng/ml) (from Bastard et al., 2021a)

Type I IFN auto-Abs in adults (and amount of type I IFN neutralized in plasma diluted 1/10)	OR [95% CI]	P value
Anti-IFN-α2 and anti-IFN-ω auto-Abs (10 ng/ml)	67.6 [4.1–1,108.6]	7.8 × 10^−13^
Anti- IFN-α2 and anti-IFN-ω auto-Abs (100 pg/ml)	54.0 [10.6–275.4]	<10^−13^
anti-IFN-α2 auto-Abs (10 ng/ml)	44.5 [8.82–225.0]	<10^−13^
anti-IFN-α2 auto-Abs (100 pg/ml)	23.3 [9.8–55.5]	<10^−13^
anti-IFN-ω auto-Abs (10 ng/ml)	12.9 [4.4–38.1]	1.4 × 10^−12^
anti-IFN-ω auto-Abs (100 pg/ml)	12.7 [7.1–22.9]	<10^−13^
anti-IFN-α2 auto-Abs only (10 ng/ml)	20.5 [3.9–107.0]	1.8 × 10^−09^
anti-IFN-α2 auto-Abs only (100 pg/ml)	9.5 [3.5–25.9]	2.8 × 10^−09^
anti-IFN-β auto-Abs only (10 ng/ml)	4.66 [0.88–24.6]	0.04
anti-IFN-ω auto-Abs only (10 ng/ml)	2.9 [0.9–9.8]	0.06
anti-IFN-ω auto-Abs only (100 pg/ml)	6 [3.1–11.7]	3.9 × 10^−10^

In comparisons of adult patients with critical COVID-19 and asymptomatic infected adult controls, ORs are higher in individuals with auto-Abs against IFN-α than in those with auto-Abs against IFN-ω only, particularly for Abs neutralizing IFN-α at a concentration of 10 ng/ml. This increase in risk for patients with auto-Abs against IFN-α is highly significant.

## Discussion

We report that auto-Abs neutralizing unglycosylated and glycosylated type I IFN-α2 or IFN-β are exceedingly rare (0.17% and 0.04%) in children from the general population, whereas about 0.35% of these children harbor auto-Abs neutralizing IFN-ω at a concentration of 10 ng/ml, and up to 2% of them harbor auto-Abs neutralizing this cytokine at 100 pg/ml. This relatively high proportion of individuals with auto-Abs neutralizing IFN-ω is similar to that found in young adults under the age of 40 years (Bastard et al., 2021a; Manry et al., 2022; van der Wijst et al., 2021). Nevertheless, all these proportions are significantly different from those for adults under the age of 70 years, in whom auto-Abs neutralizing IFN-α2 were found in only 0.3%, auto-Abs neutralizing IFN-ω were found in about 0.9%, and auto-Abs neutralizing IFN-β were found in about 0.3% (Bastard et al., 2020, 2021a; Manry et al., 2022; van der Wijst et al., 2021). Auto-Abs against glycosylated IFN-α2 and IFN-ω were not investigated in the studies on adults (Bastard et al., 2020, 2021a; Manry et al., 2022; van der Wijst et al., 2021), but the similar frequencies of auto-Abs against glycosylated and unglycosylated forms suggest that this is not a major bias. The prevalence of auto-Abs against the three type I IFNs therefore appears to remain stable over time, with two exceptions of different magnitudes. First, the levels of auto-Abs against IFN-ω decrease slightly during middle age. Second, the levels of auto-Abs against IFN-α2, and to a lesser extent IFN-ω (but not IFN-β), suddenly begin to increase after the age of 65 years (Bastard et al., 2020, 2021a; Manry et al., 2022; van der Wijst et al., 2021). The prevalence of auto-Abs neutralizing IFN-α2 increased eightfold after the age of 65 years (the increase was even as great as 10-fold in men), a much greater increase than was observed for the prevalence of auto-Abs neutralizing IFN-ω only (which increased only 2.5-fold). Overall, auto-Abs neutralizing 1 ng/ml glycosylated IFN-β are exceedingly rare in children (0.04%), rare in adults below 65 years of age (0.3%), and not more common in the elderly (0.18%). Auto-Abs neutralizing at least 100 pg/ml glycosylated IFN-α2 are very rare in children (0.17%), rare in adults (0.3%), and much more common in the elderly (2.6%). Finally, auto-Abs neutralizing at least 100 pg/ml IFN-ω are less rare in children (2%) than in adults (0.9%) and display a lesser increase in prevalence in the elderly (2%).

The levels of auto-Ab against type I IFNs do not appear to be markedly lower in the youngest children (those under the age of 5 years). This suggests that the production of these pathogenic auto-Abs, especially those against IFN-α2 or IFN-β, in children and young adults may have a germline genetic etiology. In support of this hypothesis, several inborn errors are known to underlie the occurrence of these auto-Abs. Indeed, (i) most, if not all patients with APS-1 and biallelic deleterious variants of *AIRE* produce such auto-Abs from early childhood onward, as do some patients with dominant-negative variants of *AIRE* (Ahonen et al., 1990; Bastard et al., 2021c; Meyer et al., 2016; Oftedal et al., 2015, 2023; Ossart et al., 2018); (ii) about a third of women suffering from incontinentia pigmenti due to heterozygosity for loss-of-function mutations of *IKBKG* harbor these auto-Abs (Bastard et al., 2020; Rosain, 2023); (iii) most patients heterozygous for *NFKB2* variants that are gain-of-function for IκBδ activity and loss-of-function for p52 activity, and patients with recessive deficiencies of NIK or RELB have such auto-Abs (Le Voyer, 2023); and (iv) patients with autosomal dominant IKZF2 (Helios) deficiency (Hetemäki et al., 2021a), biallelic *RAG1* or *RAG2* hypomorphic variants (Chen et al., 2014; Walter et al., 2015) or FOXP3 deficiency (Rosenberg et al., 2018) also carry these auto-Abs. New inborn errors underlying the production of these auto-Abs are expected to be discovered in the future. Other germline genetic etiologies may underlie the production of auto-Abs against IFN-α or IFN-ω, particularly in children and young adults. It is also surprising that the prevalence of anti-IFN-ω auto-Abs seems to decrease slightly with age, before increasing again, together with the prevalence of anti-IFN-α auto-Abs, after the age of 65 years. Auto-Abs arising after the age of 65 years are less likely to be caused by germline variants; their production may be due to somatic variants, epigenetic changes in hematopoietic or non-hematopoietic cell lineages, or thymic lesions, such as thymomas (Cheng et al., 2010; Meager et al., 2003; Rapnouil et al., 2023, *Preprint*; Shiono et al., 2003). The differences in prevalence with age also suggest that the pathogenesis of auto-Abs against type I IFNs may differ, in each age group, between auto-Abs neutralizing IFN-α2, IFN-β, and IFN-ω.

We also report that at least 10% of the children hospitalized for COVID-19 pneumonia studied had neutralizing auto-Abs against type I IFNs, as reported by other groups in smaller cohorts (Abolhassani et al., 2022). We also show that these auto-Abs neutralized normally glycosylated IFN-α2a/b, IFN-α14, IFN-ω, and IFN-β. Only glycosylated IFN-β was tested in our previous studies (Bastard et al., 2020, 2021a, 2021b, 2021c, 2022). The risk of life-threatening COVID-19 pneumonia in children with auto-Abs neutralizing type I IFNs is very high, as previously reported for adults (Barcenas-Morales et al., 2016; Bastard et al., 2021a; Puel et al., 2022). The very high risk of life-threatening COVID-19 pneumonia in children harboring auto-Abs against type I IFNs is consistent with that in children with recessive IEI affecting the type I IFN pathway (Zhang et al., 2022b). In the light of our screening of uninfected children, for the combinations tested, auto-Abs against IFN-α conferred a significantly higher risk of life-threatening COVID-19 than auto-Abs against IFN-ω, regardless of the concentration of cytokine neutralized. A similar pattern has also been observed in adults. The risk of critical COVID-19 in adults with auto-Abs neutralizing IFN-α (regardless of their ability to neutralize IFN-ω) is much higher than that in adults with auto-Abs neutralizing IFN-ω only (P = 0.008 at 100 pg/ml and P *=* 0.0006 at 10 ng/ml; Table 5). Furthermore, in adults with critical COVID-19 pneumonia, the prevalence of auto-Abs neutralizing IFN-α2 at 10 ng/ml doubles after the age of 60 years (5.6% before 60 years versus 11.2% after 60 years).

As previously observed in adults (Manry et al., 2022), the risk of life-threatening COVID-19 is also higher for children carrying auto-Abs neutralizing high concentrations of IFN-α2 and/or IFN-ω than for children carrying only auto-Abs neutralizing low concentrations, further suggesting that auto-Abs neutralizing high concentrations of IFN-α2 and/or IFN-ω have a more deleterious impact on COVID-19 outcomes. The risk of life-threatening COVID-19 is even higher in patients with auto-Abs neutralizing both IFN-αs and IFN-ω, further suggesting that the IFN-α subtypes and IFN-ω may not be completely redundant in the context of COVID-19. In addition, the risk of hypoxemic pneumonia increased with the concentration of type I IFNs neutralized. The risk of other viral diseases is unclear, although severe influenza has been reported in several children with auto-Abs against type I IFNs (Walter et al., 2015; Zhang et al., 2022c). Children may have higher tonic or virus-induced type I IFN levels than adults in the tissues in which these molecules are most needed as an initial barrier, such as the naso-epithelial barrier for COVID-19 (Alfi et al., 2021; Beer et al., 2022; Hatton et al., 2021; Lopez et al., 2021; Ziegler et al., 2021).

Despite our discovery of recessive IEI of type I IFN immunity in about 10% of the children studied and of auto-Abs against type I IFNs in another 10%, the cause of severe COVID-19 pneumonia remains unexplained in most children. Other auto-Abs (against type III IFNs, for example) (Vanker et al., 2023), or other IEI, possibly, but not necessarily affecting type I IFNs, might explain these remaining cases. However, our findings already have broad clinical implications. Children hospitalized for COVID-19 pneumonia should be tested for auto-Abs against type I IFNs as targeted therapies can be proposed. When effective against the circulating strains, mAbs neutralizing the virus can be effective if administered promptly (Gupta et al., 2021), as recently shown for an IRF9-deficient child during the first wave of the epidemic (Levy et al., 2021) and other patients with IEI (Johnson et al., 2023). Antiviral compounds, such as remdesivir (Beigel et al., 2020; Gottlieb et al., 2021), molnupiravir (Jayk Bernal et al., 2021), or nirmatrelvir plus ritonavir (Hammond et al., 2022), may also be of benefit in these patients, provided that they are administered sufficiently early in the course of infection. Likewise, early recombinant IFN-β therapy may be considered to prevent the development of hypoxemic pneumonia in patients whose auto-Abs do not neutralize IFN-β (Monk et al., 2021; Vinh et al., 2021). Nasal IFN-α2b could also be considered in patients without auto-Abs or IEI affecting the response to type I IFNs (Zhou et al., 2023). Treatment with type III IFNs is another possibility (Sokal et al., 2023). In the most severe cases, a combination of these therapies with plasmapheresis may be proposed (Bastard et al., 2021c).

Children with auto-Abs against type I IFNs should be followed prospectively. In the general population, it is not entirely clear which group of children should be screened because of the high risk of such auto-Abs. Children with IEI should certainly be screened, particularly those with known genetic etiologies of auto-Abs against type I IFNs (Ahonen et al., 1990; Chen et al., 2014; Oftedal et al., 2015, 2023; Walter et al., 2015; Meyer et al., 2016; Ossart et al., 2018; Bastard et al., 2021c; Hetemäki et al., 2021a; Le Voyer, 2023; Rosain, 2023). Children with a history of unusually severe viral infection should also be tested, as the clinical phenotype of anti-type I IFN auto-Ab production is expanding to include other severe viral diseases (Bastard et al., 2021b, 2022; Gervais et al., 2023; Zhang et al., 2022c). Children with auto-Abs neutralizing type I IFNs should be vaccinated against SARS-CoV-2 and influenza, but not with live-attenuated vaccines (Bastard et al., 2021b). Finally, it would be of interest to conduct pilot studies of the screening of selected populations, such as children with autoimmune conditions (e.g., lupus erythematosus, which is associated with these auto-Abs in adults) (Gupta et al., 2016; Kisand et al., 2010; Londe et al., 2023; Mathian et al., 2022; Panem et al., 1982). Many questions remain unanswered. Severe viral infections might occur at higher frequency in individuals with these auto-Abs. By inference from the known risk of severe adverse reaction to the live-attenuated virus vaccine against yellow fever in adults with auto-Abs against type I IFNs (Bastard et al., 2021b), children with these auto-Abs should not receive this vaccine. Surprisingly, MMR vaccination seems to be well tolerated in APS-1 patients despite the presence of high levels of auto-Abs against type I IFNs. Other live attenuated vaccines (against varicella-zoster virus or monkey pox, for example) should probably be avoided due to the unknown risk of adverse reaction. By contrast, children with these auto-Abs would benefit from RNA vaccination against SARS-CoV-2 and boosters as they are able to mount an Ab response capable of neutralizing the virus (Bastard et al., 2022; Sokal et al., 2023; Wolff et al., 2023). Finally, the follow-up of these children will also be of interest as the changes in the levels of these auto-Abs over time and their association with other viral, tumoral, and autoimmune diseases remain unclear. Life-long follow-up of this cohort should provide answers to these questions and help to improve the clinical management of these children.

## Materials and methods

### Study design

We enrolled 183 patients with proven COVID-19 pneumonia from nine countries (Brazil, France, Italy, Morocco, Saudi Arabia, Spain, Peru, Turkey, and Ukraine) in this study. We collected plasma or serum samples for all these individuals for immunoassay testing for the presence of auto-Abs against type I IFNs. 2,267 children from the general population were recruited in Belgium (*n* = 126), Canada (*n* = 161), Estonia (*n* = 288), Spain (*n* = 1,685), and Pakistan (*n* = 7). Two additional cohorts were established independently in Estonia and Japan, and the cohorts of patients with bacterial infections were established independently in Spain. All individuals were recruited according to protocols approved by local institutional review boards (IRBs). Written informed consent was obtained in the country of residence of each patient. Experiments were conducted in France and the United States in accordance with local regulations and with the approval of the IRB of the Institut National de la Santé et de la Recherche Médicale and the Rockefeller University, respectively. Approval was obtained from the French Ethics Committee (Comité de Protection des Personnes), the French National Agency for Medicine and Health Product Safety, the Institut National de la Santé et de la Recherche Médicale in Paris, France (protocol no. C10-13), and the Rockefeller University Institutional Review Board in New York, USA (protocol no. JCA-0700).

### COVID-19 classification

The severity of COVID-19 was assessed for each patient, as follows (Bastard et al., 2020; Zhang et al., 2020b): “critical COVID-19 pneumonia” was defined as pneumonia developing in patients with critical disease, whether pulmonary, with high-flow oxygen, mechanical ventilation (continuous positive airway pressure, bilevel positive airway pressure, intubation), septic shock, or with damage to any other organ requiring admission to the ICU. “Severe COVID-19” was defined as pneumonia developing in patients requiring low-flow oxygen (<6 L/min) supplementation.

### Statistics

OR and P values for the effect of auto-Abs neutralizing each type I IFN on critical or severe COVID-19 using patients with asymptomatic/mild disease or the general population as controls, adjusted for age in three classes (≤5 years old, (5–10 years old], and (10–18 years old]) and sex, were estimated by means of Firth’s bias-corrected logistic regression (Firth, 1993; Heinze and Schemper, 2002) as implemented in the “logistf” R package (https://rdrr.io/cran/logistf/). The risks of critical or severe COVID-19 for carriers of different combinations of neutralizing auto-Abs were compared by Firth’s logistic regression adjusted for age in three classes and sex, as described above, in the subsample of individuals (cases and individuals from the general population) carrying the auto-Ab combinations compared. The standard error of the mean for the prevalence of neutralizing auto-Abs against each type I IFN by age group and sex was estimated with the Agresti-Coull approximation (Agresti and Coull, 1998).

### Detection of anti-cytokine auto-Abs

#### Gyros

Cytokines, recombinant human (rh)IFN-α2 (ref. number 130-108-984; Miltenyi Biotec) or rhIFN-ω (ref. number SRP3061; Merck), were first biotinylated with EZ-Link Sulfo-NHS-LC-Biotin (cat. number A39257; Thermo Fisher Scientific), according to the manufacturer’s instructions, with a biotin-to-protein molar ratio of 1:12. The detection reagent contained an Alexa Fluor 647 goat anti-human IgG Ab (ref. number A21445; Thermo Fisher Scientific) diluted in Rexip F (ref. number P0004825; 1/500 dilution of the 2 mg/ml stock to yield a final concentration of 4 µg/ml; Gyros Protein Technologies). PBS-Tween (PBS-T) 0.01% buffer and Gyros Wash buffer (ref. number P0020087; Gyros Protein Technologies) were prepared according to the manufacturer’s instructions. Plasma or serum samples were then diluted 1/100 in PBS-T 0.01% and tested with Bioaffy 1000 CD (ref. number P0004253; Gyros Protein Technologies) and Gyrolab X-Pand (ref. number P0020520; Gyros Protein Technologies). Cleaning cycles were performed in 20% ethanol.

### Functional evaluation of anti-cytokine auto-Abs

#### Luciferase reporter assays

The blocking activity of anti-IFN-α2 and anti-IFN-ω auto-Abs was assessed by measuring luciferase reporter activity. Briefly, HEK293T cells were transfected with a plasmid containing the firefly luciferase gene under the control of the human *ISRE* promoter in the pGL4.45 backbone and a plasmid constitutively expressing *Renilla* luciferase for normalization (pRL-SV40). Cells were transfected in the presence of the X-tremeGene9 transfection reagent (ref. number 6365779001; Sigma-Aldrich) for 24 h. Cells in DMEM (Thermo Fisher Scientific) supplemented with 2% FCS and 10% healthy control or patient serum/plasma (after inactivation at 56°C, for 20 min) were either left unstimulated or were stimulated with IFN-α2 (ref. number 130-108-984; Miltenyi Biotec) or IFN-ω (ref. number SRP3061; Merck) at 10 ng/ml or 100 pg/ml, or with IFN-β (ref. number: 130-107-888; Miltenyi Biotec) at 10 ng/ml for 16 h at 37°C. Each sample was tested once for each cytokine and dose. Finally, cells were lysed for 20 min at room temperature and luciferase levels were measured with the Dual-Luciferase Reporter 1000 assay system (ref. number E1980; Promega) according to the manufacturer’s protocol. Luminescence intensity was measured with a VICTOR-X Multilabel Plate Reader (PerkinElmer Life Sciences). Firefly luciferase activity values were normalized against *Renilla* luciferase activity values. These values were then normalized against the median level of induction for non-neutralizing samples and expressed as a percentage. Samples were considered neutralizing if luciferase induction after normalization against *Renilla* luciferase activity was below 15% the median value for controls tested the same day.

### Phage immunoprecipitation sequencing (PhIP-Seq)

The reactivity of circulating Abs against common pathogens in plasma samples from patients and healthy controls was analyzed by PhIP-Seq, as previously described (Hasan et al., 2021). Pooled human plasma for IVIg (Privigen CSL Behring AG), human IgG-depleted serum (supplier no. HPLASERGFA5ML; Molecular Innovations, Inc.), and plasma samples from unrelated healthy children were included as controls. PhIP-Seq was carried out as previously described but with the following modifications. Total IgG levels were determined with the Human IgG total ELISA Ready-SET-Go kit (Thermo Fisher Scientific) and diluted samples containing 4 mg total IgG were incubated at 4°C overnight with 2 × 10^10^ plaque-forming units of a modified version of the original VirScan phage library. Specifically, the T7 phage library used here for peptide display contained the same viral peptides as the original VirScan phage library plus additional peptides derived from protein sequences of various microbial B cell antigens available from the Immune Epitope Database (https://www.iedb.org). For the computational analysis and background correction, the phage library was sequenced before (input library sample) and after immunoprecipitation with beads alone (mock IP). Single-end sequencing was performed with the NextSeq500 system (Illumina) to generate ∼2 million reads per sample and ∼20 million reads for the input library samples. Reads were mapped onto the original library sequences with Bowtie 2 and read counts were adjusted according to library size. A zero-inflated generalized Poisson model was used to estimate the P values to reflect enrichment for each of the peptides. We considered peptides to be significantly enriched only if the −log_10_ P value was at least 2.3 in all replicates. Species-specific score values were computed for each serum or plasma sample by counting the significantly enriched peptides for a given species with a continuous subsequence of no more than seven residues, the estimated size of a linear epitope, in common. We corrected for the nonspecific binding of peptides to the capture matrix by also calculating species-specific background score values by counting the peptides displaying enrichment to the 90th percentile for the mock IP samples. These peptides were used for background subtraction.

### Online supplemental material

Fig. S1 describes neutralizing auto-Abs against type I IFNs in children with life-threatening COVID-19. Fig. S2 describes neutralizing auto-Abs against IFN-α2 and/or IFN-ω in children and adults with life-threatening COVID-19. Fig. S3 describes neutralizing auto-Abs against glycosylated type I IFNs, and proportion of children and adults from the general population with neutralizing auto-Abs against type I IFNs. Fig. S4 describes neutralizing auto-Abs against IFN-α2 or IFN-ω in children from the general population. Fig. S5 describes neutralizing auto-Abs against IFN-α2 and/or IFN-ω in children from the general population in Estonia and Japan.

## Data Availability

The data are available from the corresponding author upon reasonable request. All the data needed to evaluate the conclusions of the paper are present in the paper or the online supplemental material.

## Data availability

The data are available from the corresponding author upon reasonable request. All the data needed to evaluate the conclusions of the paper are present in the paper or the online supplemental material.
